# eIF2α phosphorylation is required to prevent hepatocyte death and liver fibrosis in mice challenged with a high fructose diet

**DOI:** 10.1186/s12986-017-0202-6

**Published:** 2017-08-01

**Authors:** Woo-Gyun Choi, Jaeseok Han, Ji-Hyeon Kim, Mi-Jeong Kim, Jae-Woo Park, Benbo Song, Hee-Jeong Cha, Hye-Seon Choi, Hun-Taeg Chung, In-Kyu Lee, Tae-Sik Park, Maria Hatzoglou, Hueng-Sik Choi, Hyun Ju Yoo, Randal J. Kaufman, Sung Hoon Back

**Affiliations:** 1 School of Biological Sciences, University of Ulsan, Ulsan, 44610 Republic of Korea; 2 Soonchunhyang Institute of Med-bio Science (SIMS), Soonchunhyang University, Cheonan-si, Choongchungnam-do, 31151 Republic of Korea; 3 Degenerative Diseases Program, Sanford Burnham Prebys Medical Discovery Institute, 10901 North Torrey Pines Road, La Jolla, CA 92037 USA; 4 NGM Biopharmaceuticals, Inc., 333 Oyster Point Blvd, South San Francisco, CA 94080 USA; 5 Department of Pathology, Ulsan University Hospital, University of Ulsan College of Medicine, Ulsan, 44043 Republic of Korea; 6 Department of Internal Medicine and Biochemistry and Cell Biology, Kyungpook National University School of Medicine, Daegu, 41944 Republic of Korea; 7 Department of Life Science, Gachon University, Seongnam, Republic of Korea; 8 Department of Nutrition, Case Western Reserve University School of Medicine, Cleveland, OH 44106 USA; 9 School of Biological Sciences and Technology, Chonnam National University, Gwangju, Republic of Korea; 10 Biomedical Research Center, Asan Medical Center, College of Medicine, University of Ulsan, Seoul, 05505 Republic of Korea

**Keywords:** Nonalcoholic fatty liver disease, Fibrosis, High fructose diet, eIF2α phosphorylation, Aging, Oxidative stress, Antioxidant enzymes, NADPH, Glutathione

## Abstract

**Background:**

Dietary fructose can rapidly cause fatty liver in animals through de novo lipogenesis (DNL) and contribute to the development and severity of nonalcoholic fatty liver disease (NAFLD). In response to diverse cellular insults including endoplasmic reticulum (ER) and oxidative stress, phosphorylation of the eukaryotic translation initiation factor 2 alpha subunit (eIF2α) attenuates general translation initiation, allowing cells to conserve resources and initiate adaptive gene expression to restore homeostasis. The present study aimed to investigate the role of eIF2α phosphorylation in protecting against NAFLD induced by high fructose ingestion in a hepatocyte-specific eIF2α-phosphorylation-deficient mouse model.

**Methods:**

Hepatocyte-specific non-phosphorylatable (S51A) eIF2α knock-in (*A/A;fTg/0;Cre* ^*Hep*^ */0*, *A/A* ^*Hep*^) mice were generated by crossing *A/A;fTg/fTg* mice with the *floxed* WT *eIF2α* transgene (*fTg*) with *Alfp-Cre* recombinase transgenic *S/A;Cre* ^*Hep*^ */0* (*S/A-Cre* ^*Hep*^) mice. Hepatocyte-specific eIF2α-phosphorylation-deficient 3-month-old mice or 12-month-old mice were fed a 60% high fructose diet (HFrD) for 16 or 5 wks, and the effects of eIF2α-phosphorylation deficiency on NADP/NADPH and GSSG/GSH levels, ROS-defense gene expression, oxidative damage, cell death, and fibrosis were observed.

**Results:**

Prolonged fructose feeding to mice caused dysregulation of the unfolded protein response (UPR) sensor activation and UPR gene expression, and then led to decreased expression of several ROS defense genes including glutathione biogenesis genes. Nonetheless, these changes were not sufficient to induce the death of eIF2α phosphorylation-sufficient hepatocytes. However, there was a substantial increase in hepatocyte death and liver fibrosis in fructose-fed middle-aged mice deficient in hepatocyte-specific eIF2α phosphorylation because of diminished antioxidant capacity due to reduced expression of antioxidant enzymes (GPX1 and HO-1) and lower NADPH and glutathione levels, as well as a possible increase in ROS-induced damage from infiltrating NOX2-expressing leukocytes; all this led to a vicious cycle of hepatocyte death and leukocyte infiltration.

**Conclusion:**

Our findings suggest that eIF2α phosphorylation maintains NADPH and GSH levels and controls the expression of ROS-defense genes, thereby protecting hepatocytes from oxidative stresses induced by fructose metabolism.

**Electronic supplementary material:**

The online version of this article (doi:10.1186/s12986-017-0202-6) contains supplementary material, which is available to authorized users.

## Background

Nonalcoholic fatty liver disease (NAFLD) is classified into simple hepatic steatosis and nonalcoholic steatohepatitis (NASH), which may progress to liver cirrhosis and hepatocellular carcinoma (HCC). NASH has several of the characteristics of steatosis together with lobular inflammation and hepatocellular ballooning [1]. Although the pathogenesis of NAFLD/NASH is believed to involve a multistep process that begins with excess accumulation of lipids in the liver, the “two-hit” hypothesis proposed by Day et al. [2] is widely accepted. In this model the first “hit” is hepatic triglyceride accumulation or steatosis owing to abnormal hepatic lipid metabolism, which results in excessive lipid influx, decreased lipid clearance or both. The second “hit” involves inflammatory cytokines/adipokines, bacterial endotoxins, oxidative stress, mitochondrial dysfunction and/or endoplasmic reticulum stress, leading to steatohepatitis and fibrosis [3, 4].

Dietary foods are especially important contributors to the first “hit”, generating intrahepatic lipid. Among dietary foods there is much attention on fructose-sweetened foods because of the unique characteristics of this monosaccharide. Most dietary fructose is metabolized exclusively in hepatocytes because its metabolism is highly dependent on ketohexokinase (KHK), which is a hepatocyte-specific enzyme also known as fructokinase. This enzyme converts fructose to fructose-1-phosphate and is the rate-limiting enzyme in the glycolytic pathway metabolizing fructose [5, 6]. Fructose stimulates de novo lipogenesis causing hepatic steatosis via the enhanced activity of regulatory proteins involved in lipid biosynthesis, such as sterol regulatory element-binding protein-1 [SREBP-1] and carbohydrate-response element-binding protein [ChREBP] [5, 7]. Furthermore, this lipogenic carbohydrate may also cause several kinds of hepatic stresses (the second “hit”) including minimal inflammation [3, 8], oxidative stress [3, 9], mitochondrial dysfunction [3, 10], and endoplasmic reticulum (ER) stress [11, 12], so leading to steatohepatitis. Therefore, dietary fructose is believed to be a major risk factor for NAFLD, which is associated with insulin resistance and metabolic syndrome (obesity, hyperlipidemia, type 2 diabetes, and high blood pressure) [5, 7, 13].

Eukaryotic translation initiation factor 2 alpha (eIF2α) is a subunit of the eIF2 complex, which mediates binding of the initiator methionyl-tRNA (Met-tRNAi) to the ribosome during the initiation of translation of all cytoplasmic mRNAs in eukaryotic cells [14]. eIF2α is phosphorylated on serine 51 by four mammalian protein kinases, PERK, GCN2, PKR, and HRI in response to diverse cellular stresses including endoplasmic reticulum (ER) stress, amino acid deficiency, viral infection, heme deficiency, oxidative stress, etc. [15, 16]. Eukaryotic cells cope with ER stress by activating the unfolded protein response (UPR) which inhibits global protein synthesis and induces the transcription of genes encoding ER chaperones and ERAD components to enhance the capacity of productive folding and degradation mechanisms, respectively [17, 18]. Phosphorylation of eIF2α attenuates general translation initiation, allowing cells to conserve resources and initiate adaptive gene expression to restore cellular homeostasis [15, 16]. Therefore, eIF2α phosphorylation deficiency during cellular stress can disturb the control of translation initiation, leading not only to unregulated translation of general mRNAs but also to absence of translation of specific mRNAs such as that encoding ATF4, a transcriptional activator of genes involved in amino acid metabolism, cellular redox status, protein synthesis, and apoptosis [17–21]. Furthermore, eIF2α phosphorylation is also required during ER stress for activation of the ER stress sensor ATF6α by facilitating its intramembrane proteolysis [22, 23] and for maximal induction of spliced XBP1 (XBP1s) protein by stabilizing their mRNA [24]; XBP1s is a transcription factor that promotes the induction of UPR genes, which enhance ER processing capacity (protein folding and degradation) and alleviate cellular injury [18]. Thus, eIF2α phosphorylation during cellular stress is responsible for transcriptional and translational reprogramming to protect the stressed cells. Deficiency of eIF2α phosphorylation and downstream signaling pathways in in vitro or in vivo models increases sensitivity to cellular stresses including ER stress [25, 26], glucose amino acid deficiency [25, 27, 28], and oxidative stress [19, 29–31], although its phosphorylation can paradoxically induce cell death via strong and persistent induction of proapoptotic genes such as ATF4 and CHOP [21, 32, 33].

Recently, it has been proposed that the hepatic steatosis induced by dietary fructose in animal models is associated with activation of the IRE1 and PERK branches of the UPR in liver tissues [11, 12]. In addition, ATF4 and XBP1 deficiencies attenuate fructose-induced lipogenesis in the liver, possibly through independent pathways [34, 35]. Therefore, ATF4-deficient mice are protected against the development of steatosis and hypertriglyceridemia in response to high fructose feeding. Fructose-fed XBP1 knock-out mice are also protected against hepatic insulin resistance. However, further investigation is needed to identify the roles of ATF4 and XBP1 in the pathogenesis of hepatic steatosis and the biological meaning of UPR branch activation.

Several groups have reported that the eIF2α phosphorylation signaling pathway is implicated in resistance to oxidative stress [19, 29] by increasing intracellular cysteine and GSH synthesis via the transcriptional activity of ATF4 [30, 36], whose expression is translationally up-regulated by eIF2α phosphorylation [20]. In addition, short-term administration of a fructose-rich diet to normal rats induces significant changes in the expression and activity of antioxidant (SOD1/2, Catalase and GPX) [37] and prooxidant (NADPH oxidase) enzymes [38]. Therefore, the aim of the current study was to investigate the role of eIF2α phosphorylation in the pathogenesis of hepatic steatosis induced by a high fructose diet.

## Methods

### Animals and diets

The *A/A;fTg/0* mice (mixed background of C57BL/6 J and SJL), *S/A* mice (backcrossed onto a C57BL/6 J background for more than 10 generations) and *Alfp-Cre* mice (C*re* ^*Hep*^ */0,* mixed background of C57BL/6 J and CD-1) have been previously described [25, 29, 39]. Crossing of *A/A;fTg/0* mice resulted in *A/A;fTg/fTg* mice, homozygous for both the *eIF2α-Ser51Ala* mutant allele (*A*) and the *loxP*-flanked wild-type *eIF2α* transgene (*fTg*). *S/A;Cre* ^*Hep*^ */0* mice were generated by crossing heterozygous *S/A* mice with *Cre* ^*Hep*^ */0* mice. *A/A;fTg/fTg* (*A/A-fTg*) mice were crossed with *S/A;Cre* ^*Hep*^ */0* (*S/A-Cre* ^*Hep*^) mice to produce *A/A;fTg/0;Cre* ^*Hep*^ */0* (*A/A* ^*Hep*^) mice, comprising *S/A;fTg/0* (*S/A-fTg*), *S/A;fTg/0;Cre* ^*Hep*^ */0* (*S/A* ^*Hep*^), and *A/A;fTg/0* (*A/A-fTg*) genotypes (Additional file 1a). Genotypes were identified by PCR of tail DNAs with the primer pairs given in Additional file 2: Table S2. The animals were housed at 22 ± 2 °C in 55 ± 5% relative humidity with a 12-h light/dark cycle. A standard rodent chow (Purina® or LabDiet® Formula Diet #5001) or a 60% fructose diet (HFrD, Harlan Lab. Diet #TD.89247) was given ad libitum.

### Chemicals

Tunicamycin was purchased from EMD Millipore (Germany) and hydrogen peroxide, menadione, propidium iodide (PI), Hoechst 33,258, and dihydroethidine hydrochloride (DHE) were from Sigma-Aldrich.

### Antibodies

From Sigma-Aldrich: Tubulin (T5168). From Santa Cruz Biotechnology: Bcl_2_ (sc-7382), eIF2α (SC-11386), CHOP (GADD153, SC-793), and Lamin A/C (sc-6215). From Cell Signaling Technology: Bcl-XL (#2764), Bak (#3814), Bax (#2772), cleaved-caspase 3 (#9661, #9664), IRE1α (#3294), and PERK (#3192S). From Enzo Life Sciences: KDEL (ADI-SPA-827), SOD1 (ADI-SOD-100), SOD2 (ADI-SOD-111), and HO-1 (ADI-OSA-150). From EMD Millipore: COL1A1 (AB765P). From R&D systems: GPX1 (AF3798). From BD Biosciences: XIAP (610762). From Biolegend: XBP1 (919502), From Invitrogen: P-eIF2α (44728G). From Clontech: EGFP (632381). From Abcam: α-SMA (ab5694), GSR1 (ab16801), and GSS (ab91591). From Proteintech: ATF4 (10835-1-AP). Anti-ATF6α antibody was provided by Dr. Ann-Whee Lee (Weill Cornell Medical College, USA). Secondary peroxidase-conjugated antibodies were purchased from Thermo Fisher Scientific or Jackson ImmunoResearch.

### Tunicamycin injection and ALT and AST measurements

Tunicamycin (Tm, 1 mg/kg body weight) in 150 mM dextrose was injected intraperitoneally. Based on established humane endpoints (see Ethics approval and consent to participate), the liver tissues and sera from anesthetized mice were isolated at the indicated times. The activities of serum alanine transaminase (ALT) and aspartate transaminase (AST) were measured with commercially available kits (CF1002 and CF1000, IVD Lab).

### Microscopy and image analysis

Liver tissues were isolated, fixed with 10% buffered formalin, and embedded in paraffin. Sections were prepared and either stained with hematoxylin and eosin (H&E) for visualization of hepatocyte morphology by light microscopy, or subjected to other histological procedures.

To assess fibrosis, the formalin-fixed liver sections were stained with Masson’s trichrome or picrosirius red, or subjected to immunofluorescence staining using rabbit anti-collagen type I (COL1A1, EMD Millipore) and Alexa 594-conjugated goat anti-rabbit secondary antibody (Molecular Probes). Light microscope and confocal microscope images were recorded digitally by camera.

For detection of apoptotic cells, TUNEL labeling was performed on formalin fixed liver tissue sections using an ApopTag Peroxidase In Situ Apoptosis Detection Kit (CHEMICON), as reported [29]. Formalin-fixed liver sections were also subjected to immunohistochemical staining with anti-cleaved caspase-3 antibody (Cell Signaling) using an ImmPRESS™ REAGENT KIT (VECTOR Laboratories). The sections were then counterstained with Mayer’s haematoxylin (Sigma-Aldrich).

For detection of nitrotyrosine or 4-HNE, formalin fixed liver sections were subjected to immunohistochemical staining with anti-nitrotyrosine (Upstate) or anti-4-HNE (abcam) antibodies as described above.

For quantification of TUNEL-positive, cleaved caspase-3-positive, nitrotyrosine-positive or 4-HNE-positive hepatocytes, whole images of liver sections were captured at 400× magnification using a whole slide imaging system (dotSlide; Olympus) at the UNIST-Olympus Biomed Imaging Center. The entire areas of liver sections were measured using dotSlide software. The positive cells in the images were observed and quantified with image- viewing software (OlyVIA; Olympus).

To assess levels of ROS, freshly prepared frozen sections were incubated with 5 μM dihydroethidine hydrochloride (DHE, Sigma-Aldrich) for 30 min at 37 °C, after which they were observed by Olympus confocal microscopy (EX/EM = 518/605 nm), and photographed. In the presence of O_2_^−^, DHE is converted to the fluorescent molecule, ethidium, which then labels nuclei by intercalating with DNA.

For transmission electron microscope (TEM) analysis, livers were fixed with 2.5% glutaraldehyde in 0.1 M phosphate salt buffer (pH 7.4). Samples were further processed and imaged as previously described. [40].

For NAFLD activity scoring (NAS), H&E stained liver sections were examined, using a histological scoring system for NAFLD, by an experienced pathologist blinded to the genotype and experimental conditions of the samples. Briefly, the NAFLD activity score (NAS) was obtained by summing scores for steatosis (0–2), fibrosis (0-3), lobular inflammation (0–2), portal inflammation (0–2), and hepatocellular ballooning (0–2). NASH was defined in cases with NAS of ≥5.

The scale bars with scales (μm) have been inserted into the microscopic images.

### Quantitative RT-PCR

Total RNAs were isolated from liver tissues and cultured hepatocytes using Trizol reagent (Life Technologies). cDNA was prepared with a High Capacity cDNA RT kit (Ambion, Life Technologies) for semi-quantitative PCR by standard methods or for quantitative real-time (RT)-PCR normalized to the levels of *β-actin* as previously described [29]. Primer sequences are given in Additional file 2: Table S2.

### Western blot analysis

Liver tissues and cells were homogenized in Nonidet P40 lysis buffer (1% NP40, 50 mM Tris-Cl pH 7.5, 150 mM NaCl, 0.05% SDS, 0.5 mM Na-vanadate, 100 mM NaF, 50 mM β-glycerophosphate, 1 mM PMSF) supplemented with Halt Protease Inhibitor Cocktail (Thermo Fisher Scientific) as described [41]. Homogenates were centrifuged at 12,000 g for 15 min at 4 °C, and supernatants were collected. Liver nuclear extracts were prepared using an NE-PER™ Nuclear and Cytoplasmic Extraction Kit (Thermo Fisher Scientific). Liver lysates, nuclear extracts, or cell lysates were used for Western blotting as described [42].

### Triglyceride measurement

Serum and liver triglyceride levels were measured with a colorimetric assay kit (TG-S AM157, ASAN Pharmaceutical). Total liver lipids were extracted with a chloroform-methanol (2:1, vol/vol) mixture according to the Folch method [43].

### Cell culture and treatments

Immortalized wild-type (HEP *S/S*) and homozygous mutant (HEP *A/A*) hepatocytes were cultured as described previously [29]. Primary mouse hepatocytes were obtained by liver perfusion with collagenase IV (Sigma Aldrich, C5138), inoculated on collagen-coated plates with/without round coverslips (5 × 10^5^ cells/well in 6-well plates), and cultured in Dulbecco’s modified Eagle’s medium (DMEM, high glucose) medium containing 10% FBS, and penicillin/streptomycin. The medium was replaced with FBS-free DMEM media 2 h after plating and the hepatocytes were incubated for a further 12 h before experimental treatment. Drugs were added in fresh DMEM.

### Cell viability analysis

For primary hepatocytes, PI and Hoechst staining viability assays were performed as previously described with slight modifications [44]. Drug-treated primary hepatocytes on coverslips were double-stained with propidium iodide (PI, 1 μg/ml) and Hoechst 33,258 (1 μg/ml), fixed with 3.5% (*w*/*v*) paraformaldehyde (Sigma Aldrich) at room temperature for 15 min, and the coverslips were mounted on glass slides glasses for observation by fluorescence microscopy. Propidium iodide is a vital nucleic acid-staining dye that penetrates cells with compromised plasma membranes (necrotic cells). Morphological changes in the nuclei of cells undergoing apoptotic cell death were determined by staining with the DNA-binding fluorochrome Hoechst 33258. Apoptotic nuclear changes include condensation, margination, and segmentation of the nuclei into several fragments. Quantitation of dead cells (apoptotic and necrotic cells) was performed by counting at least 500 cells and was expressed as a percentage of total cells counted.

### Flow cytometric analysis

Cells were loaded with CM-H2DCFFDA (Molecular Probe) for 30 min followed by recovery for 30 min before fluorescence measurements. A FACS Caliber (BD) and Flowjo software were used for the analysis.

### Measurement of hepatic reduced (GSH) and oxidized (GSSG) glutathione content

Tissue levels of GSH and GSSG content were measured using a GSSG/GSH Quantification Kit (Dojindo Molecular Technologies).

### Measurement of hepatic NADPH and NADP contents by liquid chromatography-tandem mass spectrometry (LC-MS/MS)

Standard metabolites (NADP and NADPH) were purchased from Sigma-Aldrich. All solvents including water were purchased from J. T. Baker.

10-15 mg of liver tissue was homogenized using a TissueLyzer (Qiagen) with 400 μL of chloroform/methanol (2/1). The homogenates were incubated for 20 min at 4 °C. Glutamine-d_4_ (surrogate internal standard) was added after incubation, and mixed well. Then each sample was centrifuged at 13000 rpm for 10 min. The supernatant was collected and 100 μL of H_2_O was added. The sample was mixed vigorously and centrifuged at 4000 rpm for 20 min. The upper phase was taken and dried under vacuum. The dried sample was stored at −20 °C and reconstituted with 40 μL of H_2_O/acetonitrile (50/50 *v*/v) prior to LC-MS/MS analysis. An LC-MS/MS equipped with 1290 HPLC (Agilent), Qtrap 5500 (ABSciex), and a reverse phase column (Synergi fusion RP 50 × 2 mm) was used. 5 mM ammonium acetate in H_2_O and 5 mM ammonium acetate in acetonitrile were used as mobile phases A and B, respectively. The separation gradient was as follows: hold at 0% B for 5 min, 0% to 90% B for 2 min, hold at 90% for 8 min, 90% to 0% B for 1 min, then hold at 0% B for 9 min. LC flow rate was 70 μL/min except 140 μL/min between 7 and 15 min at 23 °C. Multiple reaction monitoring (MRM) was used in negative ion mode, and the area under the curve of each EIC was normalized to that of the EIC (extracted ion chromatogram) of the internal standard, and the ratio was used for comparisons.

### Statistical analysis

All data are presented as means ± SEM. The statistical significance of differences between groups was evaluated using Student’s t test or the ANOVA one-way test (Tukey’s test). *p* < 0.05 was considered significant. ^*, #, &^ *p* < 0.05, **^, ##, &&^ *p* < 0.01, ***^, ###, &&&^ *p* < 0.001.

## Results

### eIF2α phosphorylation in hepatocytes is not required for survival of adult mice

Previously, we described a unique mouse model in which death due to homozygous Ser51Ala mutations that prevent eIF2α phosphorylation was rescued by ubiquitous expression of wild-type eIF2α in a floxed *eIF2α* transgene (*fTg*) [29]. Here, this model was used to investigate the physiological roles of eIF2α phosphorylation in hepatocytes. We generated a mouse model through the *Cre-LoxP* system, of genotype *A/A;fTg/0;Cre* ^*Hep*^ */0* (*A/A* ^*Hep*^) (Additional file 1a), unable to phosphorylate eIF2α in hepatocytes.

To confirm that eIF2α phosphorylation was sufficiently reduced by Cre-mediated recombination in the hepatocytes of the *A/A* ^*Hep*^ mice, levels of the *floxed eIF2α* transgene (*fTg*) and total *eIF2α* mRNA in total liver RNAs (Additional file 1b) were analyzed along with total and phosphorylated eIF2α proteins in total liver lysates (Additional file 1c and d). The amount of floxed transgene (*fTg*)-derived *eIF2α* mRNA in liver tissues of *A/A* ^*Hep*^ mice was decreased to ~2% of that detected in liver tissue of *S/A-fTg* (*Cont.*) and *A/A-fTg* mice (Additional file 1b, left panel). Consistent with this, the ~9-fold increase in total *eIF2α* mRNA (due to *fTg* mRNA expression) was reduced to the endogenous level by deletion of *fTg* (Additional file 1b, right panel). Western blot analysis of liver tissues revealed that total eIF2α protein expression was increased by only 1.4-fold in *A/A-fTg* livers compared to *S/A* livers, and that the high level of eIF2α protein in *A/A-fTg* was reduced to the endogenous level by *fTg* deletion (Additional file 1c). Furthermore, no phosphorylated eIF2α (p-eIF2α) was detected in liver lysates of a chemical ER stress inducer tunicamycin (Tm)-challenged *A/A* ^*Hep*^ mice, but EGFP was highly expressed due to deletion of the *floxed eIF2α* region of the transgene which activates *Egfp* expression (Additional file 1d). However, both eIF2α phosphorylation and expression of the downstream factor, CHOP, were detected in liver lysates of Tm-challenged *Control* (*Cont.*) mice (Additional file 1d). Collectively, these results demonstrate that the floxed wild-type *eIF2α* transgene in *A/A* ^*Hep*^ hepatocytes was efficiently deleted by CRE-mediated recombination.

Since the homozygous Ser51Ala mutation in the phosphorylation site of eIF2α causes postnatal lethality due to defective hepatic gluconeogenesis [25], we expected the postnatal survival frequency of *A/A;fTg/0;Cre* ^*Hep*^ */0* (*A/A* ^*Hep*^) mice to be low because of the absence of eIF2α phosphorylation in hepatocytes. However, genotype analysis of 2- to 3-month-old offspring demonstrated that most *A/A* ^*Hep*^ mice were viable (Additional file 3: Table S1), had normal body mass (Additional file 1e), and were morphologically indistinguishable from their littermates of other genotypes (data not shown), suggesting that eIF2α phosphorylation in hepatocytes is dispensable for the development of adult mice.

### eIF2α phosphorylation is required for cells to defend against ER and oxidative stress

**Fig. 1:**
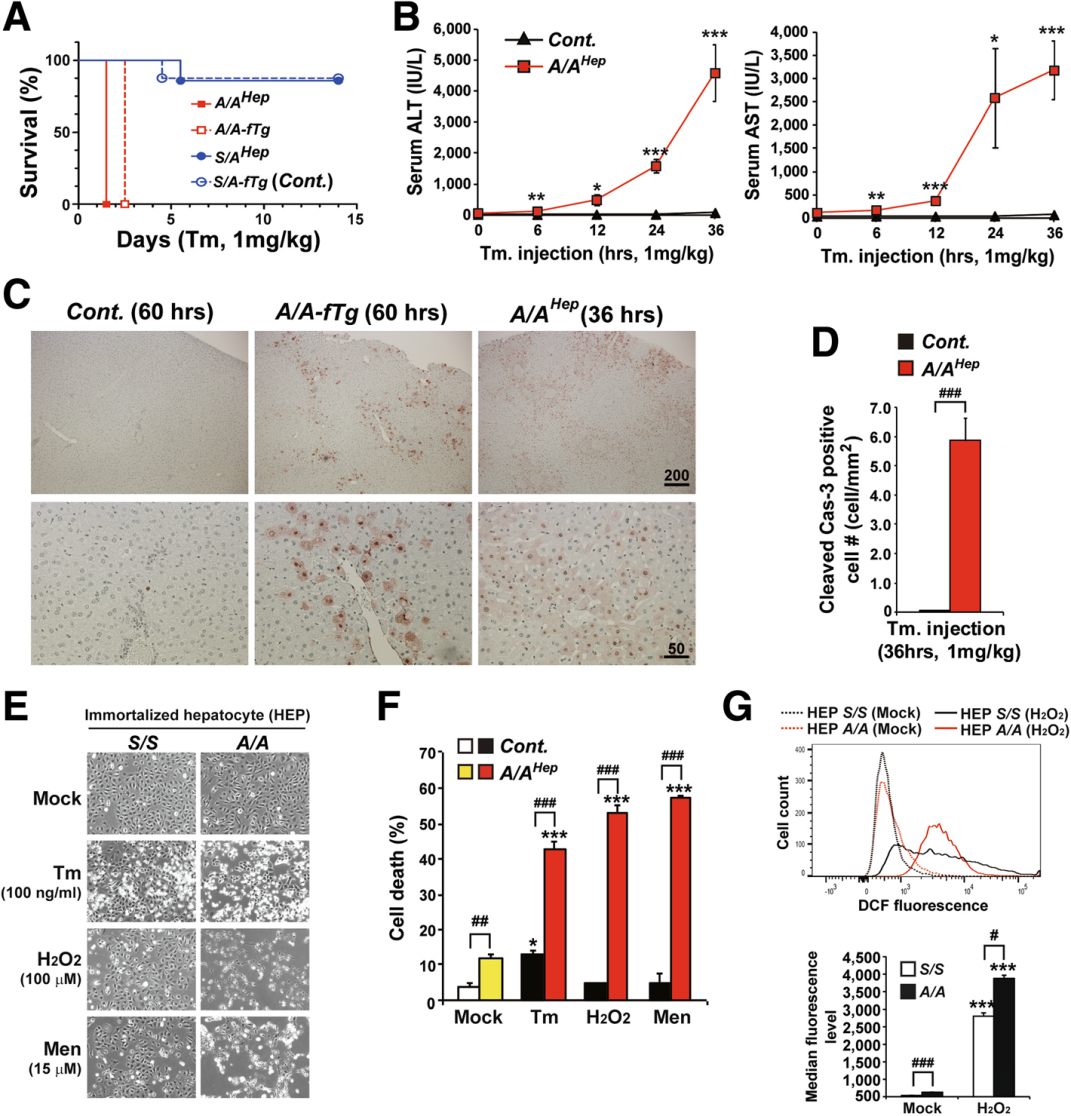
eIF2α phosphorylation protects hepatocytes from ER and oxidative stress. **a** The viability of the four experimental mouse groups (*n* = 10 per group) intraperitoneally injected with a chemical ER stress inducer (tunicamycin, Tm, 1 mg/kg body weight). Survival was followed for 14 days. **b** Serum ALT and AST levels measured at the indicated times after Tm injection. Data are means ± SEMs (*n* = 5 ~ 6 mice per group at each time point), *p < 0.05, **p < 0.01 and ***p < 0.001; *Cont.* vs *A/A* ^*Hep*^. **c** TUNEL analysis performed on liver sections obtained from mice at the indicated times after Tm injection. Scale bars with scales (μm) have been inserted into the images. Representative images are shown (*n* = 6 mice per group). **d** Quantification of cleaved caspase-3-positive cells. Liver sections of Tm-challenged animals (36 h) were stained with antibody against cleaved caspase-3. Data are means ± SEM (*n* = 4 ~ 5 mice per group), ^###^ *p* < 0.001; *Cont.* vs *A/A* ^*Hep*^. **e** Morphology of immortalized embryonic wild type (HEP *S/S*) and mutant (HEP *A/A*) hepatocytes treated with Tm (100 ng/ml, 24 h) and inducers of reactive oxygen species (ROS) H_2_O_2_ (100 μM, 10 h) and menadione (Men, 15 μM, 6.5 h) at the indicated concentrations for 6.5 ~ 24 h. Representative images are shown. **f** Cell death (apoptotic and necrotic cells) determined by double staining with Hoechst 33258 and propidium iodide. Primary hepatocytes were isolated from control (*Cont.*) and *A/A* ^*Hep*^ mice, and exposed to the indicated chemicals (5 μg/ml Tm, 700 μM H_2_O_2_ and 6 μM Men) for 24 h. At least 500 cells were counted, and cell death is expressed as a percentage of total cells. Data are means ± SEM (*n* = 3 mice per each treatment), *p < 0.05 and ***p < 0.001; Mock vs Chemicals in the same genotype, ^##^ *p* < 0.01 and ^###^ *p* < 0.001; *Cont.* vs *A/A* ^*Hep*^. **g** FACS measurement of hepatocyte ROS levels by staining with CM-H2DCFDA (*upper panel*). Wild type (HEP *S/S*) and mutant (HEP *A/A*) hepatocytes were treated with H_2_O_2_ (100 μM) for 10 h. The *lower graph* shows the average median fluorescence levels of three independent samples per group. Data are means ± SEM (*n* = 3 mice per each treatment), ***p < 0.001; Mock vs H_2_O_2_ in the same genotype, ^#^ *p* < 0.05 and ^###^ *p* < 0.001; *S/S* vs *A/A*

**Fig. 2:**
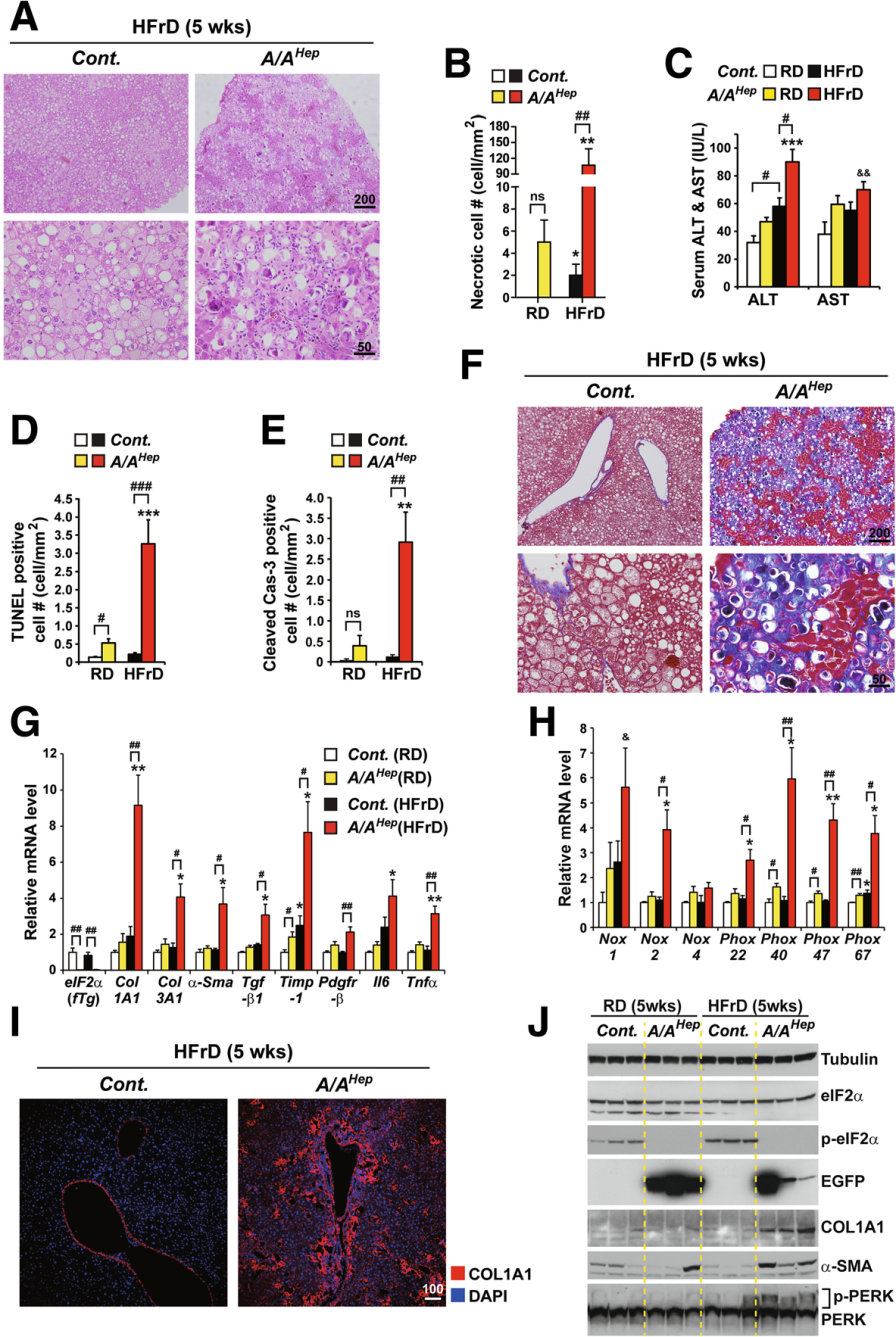
A high fructose diet (HFrD) accelerates hepatocyte cell death and fibrogenesis in hepatocyte-specific eIF2α phosphorylation-deficient middle-aged mice.** a** Hematoxylin and eosin (H&E)-stained images of liver tissue sections from 13-month-old *Cont.* and *A/A* ^*Hep*^ mice fed a 60% high fructose diet (HFrD) for 5 wks. Representative images are shown (*n* = 7 mice per group). **b** Quantification of anuclear necrotic cells in Masson’s trichrome stained liver sections (Additional file 4 e and Fig. 2f) of 13-month-old *Cont.* and *A/A* ^*Hep*^ mice fed a regular diet (RD) (*n* = 5 ~ 8 mice per group) or a 60% high fructose diet (HFrD) (*n* = 7 mice per group) for 5 wks. Data are means ± SEM; **p < 0.01; RD vs HFrD in the same genotype, ^#^ *p* < 0.05 and ^##^ *p* < 0.01; *Cont.* vs *A/A* ^*Hep*^. ns stands for no significant. **c** Serum ALT and AST levels in 13-month-old *Cont.* and *A/A* ^*Hep*^ mice fed an RD (*n* = 5 ~ 8 mice per group) or an HFrD (*n* = 7 mice per group) for 5 wks . Data are means ± SEM; ***p < 0.001; RD vs HFrD in the same genotype, ^#^ *p* < 0.05; *Cont.* vs *A/A* ^*Hep*^. **d** and (**e**) Quantification of TUNEL positive cells (**d**) and cleaved caspase 3-positive cells (**e**) in liver tissue sections (Additional file 4 b and c) from 13-month-old *Cont.* and *A/A* ^*Hep*^ mice fed an RD (*n* = 5 ~ 8 mice per group) or an HFrD (*n* = 7 mice per group) for 5 wks. Data are means ± SEM; **p < 0.01 and ***p < 0.001; RD vs HFrD in the same genotype, ^#^ *p* < 0.05, ^##^ *p* < 0.01 and ^###^ *p* < 0.001; *Cont.* vs *A/A* ^*Hep*^. ns stands for no significant. **f** Masson’s trichrome-stained images of liver tissue sections from 13-month-old *Cont.* and *A/A* ^*Hep*^ mice fed an HFrD for 5 wks. Representative images are shown (*n* = 7 mice per group). **g** and (**h**) Quantitative real-time PCR analysis of expression of selected genes ((eIF2α transgene, liver fibrosis-related genes, and inflammatory genes in Fig. 2g) and (NADPH oxidases and their components in Fig. 2h)) in livers of 13-month-old *Cont.* and *A/A* ^*Hep*^ mice fed an RD (*n* = 5 ~ 8 mice per group) or an HFrD (*n* = 7 mice per group) for 5 wks . Data are means ± SEM; *p < 0.05 and **p < 0.01; RD vs HFrD, ^#^ *p* < 0.05 and ^##^ *p* < 0.01; *Cont.* vs *A/A* ^*Hep*^ *,* ^&^ *p* < 0.05; *Cont.*(RD) vs *A/A* ^*Hep*^(HFrD). **i** Collagen AI (Col AI) immunofluorescence labeling of liver sections from 13-month-old mice fed an HFrD for 5 wks. Representative merged images of Col AI and DAPI are shown (*n* = 7 mice per group). **j** Western blot analysis of liver lysates from 13-month-old *Cont.* and *A/A* ^*Hep*^ mice fed an RD or an HFrD for 5 wks. The efficiency of deletion of *floxed eIF2α fTg* by Cre recombinase in *A/A* ^*Hep*^ livers was determined from the existence of phosphorylated eIF2α and EGFP proteins. To assess liver fibrogenesis, the levels of collagen AI (Col AI) and α-smooth muscle actin (α-SMA) in liver tissue were analyzed

As we expected, all Tm-challenged *A/A* ^*Hep*^ mice had significantly increased ALT and AST levels; at the same time there was profound hepatocyte death and the mice died after 36 h, whereas most *S/A* ^*Hep*^ and *Control* (*Cont.*) mice survived without significant hepatocyte death (Fig. 1a–d). Surprisingly, Tm-induced ER stress was also lethal to the *A/A-fTg* mice (Fig. 1a) although they did not display any difference in birth rate from their littermates of other genotypes. All the *A/A-fTg* mice died by 60 h after Tm injection and there was extensive hepatocyte death (Fig. 1a and c), suggesting that the hepatocytes were not fully functional due to insufficient wild type eIF2α being expressed from the *eIF2α* transgene (*fTg*) (Additional file 1c). Therefore, the *S/A-fTg* mice instead of the *A/A-fTg* mice were used as the control group (*Cont.*) in further experiments except those fed a regular diet in Fig. 2b –e, g, h and and Additional file 4. Thus, we conclude that eIF2α phosphorylation in hepatocytes is required for protection against ER stress.

As we and others have previously reported [21, 31], eIF2α phosphorylation-deficient immortalized hepatocytes (HEP *A/A*) and primary hepatocytes (*A/A* ^*Hep*^) were highly sensitive to reactive oxygen species (ROS)-mediated oxidative stress (H_2_O_2_ and menadione) including ER stress (Tm) compared to control cells (HEP *S/S* and *Cont.*) (Fig. 1e and f). Furthermore, when the immortalized hepatocytes were exposed to hydrogen peroxide, HEP *A/A* accumulated more elevated levels of reactive oxygen species (ROS) than HEP *S/S* (Fig. 1g), suggesting that eIF2α phosphorylation-deficient hepatocytes have reduced ROS-scavenging ability. Thus, eIF2α phosphorylation in hepatocytes is also required to protect against oxidative stress [45].

### A high fructose diet (HFrD) aggravates hepatocyte death and liver fibrosis in middle-aged *A/A*^*Hep*^ mutant mice

When given a regular diet (RD), the survival of *A/A* ^*Hep*^ mice was similar to that of control littermates (*A/A-fTg* or *S/A-fTg* mice) for more than 1 yr. However, microscopic observation of liver sections after hematoxylin and eosin (H&E) staining revealed that some 13-month-old *A/A* ^*Hep*^ mutant mice fed an RD (38%; 3 out of 8 mice, Fig. 2b and Additional file 4a) displayed increased necrotic hepatocyte death, with atypic and fragmented nuclei and strong basophilic (purple) staining. To assess hepatocyte death in 13-month-old *A/A* ^*Hep*^ mutant mice fed an RD, we measured several parameters (Necrotic cell #, Serum ALT/AST levels, TUNEL positive cell #, and cleaved casp-3 positive cell #) in the liver tissues of 13-month-old *cont.* and *A/A* ^*Hep*^ mutant mice fed an RD (Fig. 2b–e and Additional file 4a–c). When the analyzed parameters of hepatocyte death in RD-fed *A/A* ^*Hep*^ mutant mice were compared with those of RD-fed *cont.* mice, the difference of TUNEL level but not the difference of the other analytic data reaches statistical significance (Fig. 2b–e). Furthermore, histological analyses based on picrosirius red staining and Masson’s trichrome staining of hepatic collagen deposition revealed that the affected 13-month-old *A/A* ^*Hep*^ mutant mice fed an RD (3 out of 8 mice), but none of the *cont.* mice fed an RD, had hepatic fibrosis (Additional file 4d and e). Together, these data suggest that long-term deficiency of hepatocyte eIF2α phosphorylation may minimally affect cell viability and liver fibrosis development in middle-aged *A/A* ^*Hep*^ mice on the regular diet condition.

To examine whether increased de novo hepatic lipogenesis and an excess energy source affect the functioning and viability of eIF2α phosphorylation-deficient hepatocytes, 12-month-old *Cont.* and *A/A* ^*Hep*^ mutant mice were challenged with a 60% fructose diet (HFrD) for 5 wks. As we expected, HFrD feeding induced micro- and macro-vesicular steatosis in the livers of both groups (Fig. 2a). However, liver sections of the HFrD-fed *A/A* ^*Hep*^ mutant mice displayed extensive necrosis with atypic and fragmented nuclei and strong basophilic (purple) staining (Fig. 2a and b) similar to 13-month-old *A/A* ^*Hep*^ mice on a regular diet (Additional file 4a and Fig. 2b), whereas hepatocyte necrosis was not detected in the HFrD-fed *Cont.* mice. Furthermore, leukocyte infiltration was markedly increased in the livers of HFrD-fed *A/A* ^*Hep*^ mice (Fig. 2a, lower right). The increased ALT levels (Fig. 2c) and numbers of TUNEL positive cells (Fig. 2d and Additional file 4b) and activated (cleaved) caspase 3-positive cells (Fig. 2e and Additional file 4c) point to significant liver injury in the HFrD-fed *A/A* ^*Hep*^ mice. In keeping with the leukocyte infiltration, necrosis, and apoptosis, Masson’s trichrome staining indicated that liver fibrosis was markedly increased in HFrD-fed *A/A* ^*Hep*^ mutant mice (Fig. 2f). Moreover, transcripts of key genes that regulate hepatic fibrosis, collagen 1A1 and 3A1 (*Col1A1*and *Col3A1*), α-smooth muscle actin (*α-Sma*), transforming growth factor-β1 (*Tgf-β1*), tissue inhibitor of metalloproteinases 1 (*Timp-1*), and platelet-derived growth factor receptor-*β* (*Pdgfr-β*) were all increased in HFrD-fed *A/A* ^*Hep*^ mutant mice (Fig. 2g). In addition, the expression of *Il*6 and *Tnfα*, proinflammatory cytokine genes were higher in the HFrD-fed *A/A* ^*Hep*^ mutant mice(Fig. 2g).

Furthermore, transcripts of *Nox2*, the catalytic subunit of nicotinamide adenine dinucleotide phosphate oxidase and its components (*Phox22*, *40, 47,* and *67*) were significantly elevated in the livers of HFrD-fed *A/A* ^*Hep*^ mice (Fig. 2h), suggesting that phagocytes (such as Kupffer cells and neutrophils) as well as hepatic stellate cells having high levels of NOX2 and its components, which play crucial roles in hepatocyte damage [46, 47] and hepatic fibrogenesis, [48–50] had been recruited and activated. The fibrogenesis in the HFrD-fed *A/A* ^*Hep*^ mutant livers was further indicated by immunofluorescence analysis using specific antibodies against collagen type 1 A1 (COL1A1) (Fig. 2i) and immunoblotting for COL1A1 and α-smooth muscle actin (α-SMA) (Fig. 2j). As previously reported [11, 51], increased PERK phosphorylation was detected in liver lysates of both groups of HFrD-fed mice (Fig. 2j). As mentioned above, eIF2α phosphorylation was not seen in either regular diet (RD)- or HFrD-fed *A/A* ^*Hep*^ mutant mice, whereas it was increased 1.6-fold by the high fructose (HFr) diet in the *Cont.* mice. *A/A* ^*Hep*^ mice should therefore lack an HFrD-induced PERK-eIF2α signaling pathway.

In summary, hepatocyte death, inflammation, and liver fibrosis in middle-aged *A/A* ^*Hep*^ mice deficient in hepatic eIF2α phosphorylation were aggravated by an HFrD; thus eIF2α phosphorylation in hepatocytes opposes the deleterious effects of an HFrD treatment.

### eIF2α phosphorylation in adult mice is required to attenuate HFrD-induced hepatocyte death and fibrosis

To see whether high fructose (HFr) diet can induce hepatocyte cell death and liver fibrosis in adult *A/A* ^*Hep*^ mutant mice, we analyzed 7-month-old *Cont.* and *A/A* ^*Hep*^ mutant mice that were fed an HFrD for 16 wks. The HFr diet stimulates de novo lipogenesis in hepatocytes, causing lipid accumulation [52]. The body weights of both HFrD-fed *Cont.* and *A/A* ^*Hep*^ mice gradually increased up to 9 wks (Additional file 5a). As expected, there were also substantial increases in hepatic triglyceride (Additional file 5b) although serum triglyceride levels were only marginally elevated (Additional file 5c). Consistent with these changes, expression of genes (*Srebp1c*, *Acc1*, *Acc2*, *Scd1* and *Fasn*) encoding key enzymes in de novo triglyceride synthesis also increased in both groups of mice (Additional file 5d), suggesting that hepatic de novo lipogenesis is not affected by absence of eIF2α phosphorylation.

**Fig. 3:**
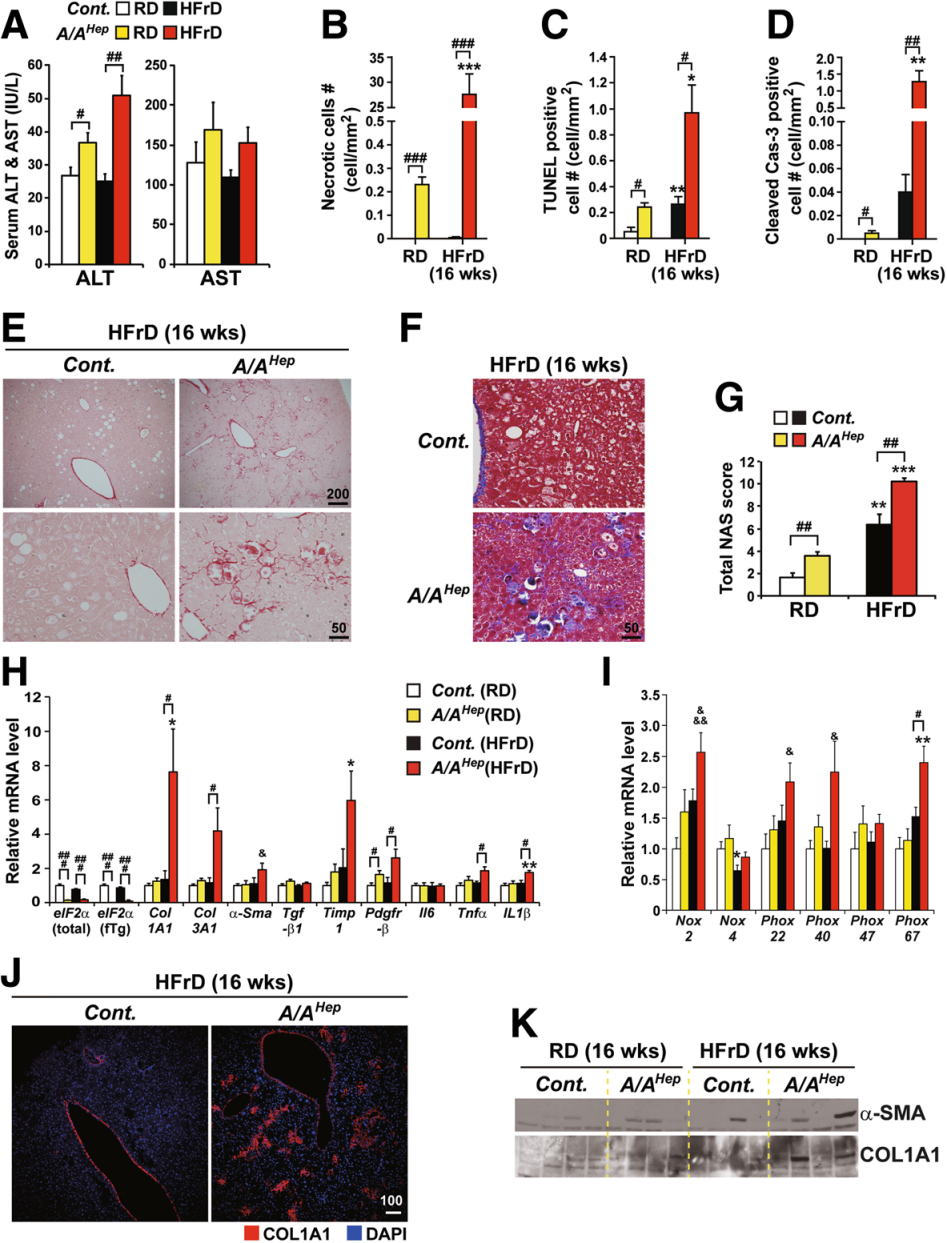
eIF2α phosphorylation in adult mice prevents hepatocyte death and hepatic fibrogenesis induced by long-term HFrD feeding. **a** Serum ALT and AST levels measured in 7-month-old *Cont.* and *A/A* ^*Hep*^ mice fed an RD or an HFrD for 16 wks. Data are shown as means ± SEM (*n* = 6 mice per group); ^#^ *p* < 0.05 and ^##^ *p* < 0.01; *Cont.* vs *A/A* ^*Hep*^. **b** Quantification of anuclear necrotic cells in the Masson’s trichrome-stained images of Fig. 4f and Additional file 6 d. Data are means ± SEM (*n* = 6 mice per group); ***p < 0.001; RD vs HFrD and ^###^ *p* < 0.001; *Cont.* vs *A/A* ^*Hep*^. **c** Quantification of TUNEL-positive cells in the images of Additional file 6 e. TUNEL staining in liver sections from 7-month-old *Cont.* and *A/A* ^*Hep*^ mice fed an RD or an HFrD for 16 wks. Data are means ± SEM (*n* = 6 mice per group); *p < 0.05 and **p < 0.01; RD vs HFrD, ^#^ *p* < 0.05; *Cont.* vs *A/A* ^*Hep*^. **d** Quantification of cleaved caspase-3-positive cells in the images of Additional file 6 f. Immunohistochemical staining of cleaved caspase 3 in liver tissue sections from 7-month-old *Cont.* and *A/A* ^*Hep*^ mice fed an RD or an 60% HFrD for 16 wks. Data are means ± SEM (*n* = 6 mice per group); **p < 0.01; RD vs HFrD, ^#^ *p* < 0.05 and ^##^ *p* < 0.01; *Cont.* vs *A/A* ^*Hep*^. **e** and (**f**) Sirius red-stained images (**e**) and Masson’s trichrome-stained images (**f**) of liver tissue sections from 7 months-old *Cont.* and *A/A* ^*Hep*^ mice fed an RD or an HFrD for 16 wks. Representative images are shown (*n* = 6 mice per group). **g** Histological NAFLD activity scores (NAS) determined according to the NASH Clinical Research Network (CRN) scoring system. Data are means ± SEM (*n* = 6 mice per group); ^##^ *p* < 0.01; *Cont.* vs *A/A* ^*Hep*^, **p < 0.01 and ***p < 0.001; RD vs HFrD. **h** and (**i**) Quantitative real-time PCR analysis of expression of selected genes (eIF2α transgene and liver fibrosis-related genes in Fig. 3h) and (NADPH oxidases and their components in Fig. 3i)) in 7-month-old *Cont.* and *A/A* ^*Hep*^ livers after 16 wk. of RD or HFrD. Data are means ± SEM (*n* = 6 mice per group); *p < 0.05; RD vs HFrD, ^#^ *p* < 0.05 and ^###^ *p* < 0.001; *Cont.* vs *A/A* ^*Hep*^, ^&^ *p* < 0.05 and ^&&&^ *p* < 0.001; *Cont.*(RD) vs *A/A* ^*Hep*^(HFrD). **j** Immunofluorescence staining of collagen AI (Col AI) in liver tissues from 7-month-old *Cont.* and *A/A* ^*Hep*^ mice fed an HFrD for 16 wks. Representative images are shown (*n* = 6 mice per group). **k** Western blot analysis of liver fibrogenesis indicators, α-smooth muscle actin (α-SMA) and collagen AI (Col AI) in liver tissues from 7-month-old *Cont.* and *A/A* ^*Hep*^ mice fed an RD or an HFrD for 16 wks

Similar to RD-fed 13-month-old *A/A* ^*Hep*^ mice (Fig. 2b–e), there was increased liver injury (ALT level, necrotic cell #, TUNEL positive cell #, and cleaved-cas3 positive cell #) in RD-fed 7-month-old *A/A* ^*Hep*^ mice although each value was relatively small (Fig. 3a–d), suggesting that absence of eIF2α phosphorylation in hepatocytes results in minimal liver injury. Since dietary high fructose is a risk factor for non-alcoholic fatty liver disease [5], which affects the functioning and viability of hepatocytes [7, 53], we measured HFrD-mediated liver injury in eIF2α phosphorylation-deficient (*A/A* ^*Hep*^) mice indirectly from the levels of serum ALT and AST. Regardless of diet, the ALT levels in *A/A* ^*Hep*^ mice were higher than in the controls, and they were further increased in the *A/A* ^*Hep*^ mice by feeding the HFr diet for 16 wks (Fig. 3a left panel). AST levels in the *A/A* ^*Hep*^ mice were also higher than in the *Cont.* mice, although the difference did not achieve statistical significance (Fig. 3a right panel). Microscopic observation of formalin fixed liver sections (Additional file 6a and b) and frozen liver sections (Additional file 6c) revealed a large number of necrotic hepatocytes (Fig. 3b and Additional file 6a right lower panel) with atypic or fragmented nuclei and strong basophilic (purple) staining (Additional file 6b). Interestingly, most of the necrotic hepatocytes were surrounded by infiltrating leukocytes (insets in Additional file 6a and c) in the liver tissues of the HFrD-fed *A/A* ^*Hep*^ mice. Moreover, hepatocyte apoptotic death was detected by the TUNEL assay in these mice (Fig. 3c and Additional file 6e), and immunohistochemistry (IHC) for cleaved caspase-3 (Fig. 3d and Additional file 6f). Thus, long-term consumption of an HFrD reduces the viability of eIF2α phosphorylation-deficient hepatocytes.

In keeping with the leukocyte infiltration and hepatocyte apoptosis and necrosis (Fig. 3b–d and Additional file 6a–c, e and f ), the livers of HFrD-fed *A/A* ^*Hep*^ mice displayed significantly increased liver fibrosis (Fig. 3e and f). Under the RD condition, *A/A* ^*Hep*^ mice already had a modestly increased total NAFLD activity score (NAS) due to some fibrosis (Fig. 3g and Additional file 6d). However, although HFrD feeding also increased the NAS score in *Cont.* mice, the score in the livers of the HFrD-fed *A/A* ^*Hep*^ mice was significantly higher, due to marked fibrosis and inflammation (Fig. 3g). In line with the hepatic fibrogenesis in HFrD-fed *A/A* ^*Hep*^ mice, there was strongly increased expression of liver fibrosis marker genes (*Col1A1*, *Col3A1*, *Timp1* and *Pdgfr-β*) in the livers of the HFrD-fed *A/A* ^*Hep*^ mice. Furthermore, the expression of proinflammatory cytokine genes (*Tnfα* and *IL1β*) (Fig. 3h) and ROS-generating NADPH oxidase genes (*Nox2*, *Phox22*, *Phox40* and *Phox67*) (Fig. 3i) was especially high in the livers of these mice, suggesting that phagocytes (such as Kupffer cells and neutrophils) and hepatic stellate cells, which play crucial roles in hepatocyte damage [46, 47] and hepatic fibrogenesis [48–50], had been recruited and activated. Hepatic fibrogenesis in the HFrD-fed *A/A* ^*Hep*^ liver tissues was further demonstrated by microscopic observation and Westerns blots showing increased expression of COL1A1 and α-SMA (Fig. 3j and k). Collectively, these HFr diet studies suggest that hepatocyte death, inflammation, and fibrosis in adult *A/A* ^*Hep*^ mice deficient in hepatic eIF2α phosphorylation were aggravated by an HFrD. In addition, since middle-aged *A/A* ^*Hep*^ mice were prone to have more liver injury than adult *A/A* ^*Hep*^ mice (Additional file 7 a) and the severity of HFrD-mediated damage was higher in the middle-aged *A/A* ^*Hep*^ mice (Additional file 7 b), we concluded that the HFrD-mediated damage seen in middle-aged *A/A* ^*Hep*^ mice was aggravated by several aspects of the aging process and/or by long-term eIF2α phosphorylation deficiency.

Consistent with the histological observations (Figs. 2a, b, d, e and 3a–d) of hepatocyte death in HFrD-fed *A/A* ^*Hep*^ liver tissues, HFrD feeding to *A/A* ^*Hep*^ mice up-regulated transcript levels of apoptosis-related genes (*Noxa* and *Casp12*) (Additional file 8 a) [54]. Furthermore, different transcripts of antiapoptotic (*Bcl2* and *Bcl-xl*) and proapoptotic (*Bak*, *Bax, Bim, Bid, Puma,* and *Noxa*) genes were up or downregulated (Additional file 8 a) but the changes were <1.5-fold except for *Noxa*. However, Western blot analysis revealed that the proapoptotic protein Bax was significantly higher in livers of HFrD-fed *A/A* ^*Hep*^ mice than in those of RD- or HFrD-fed *cont.* mice, even though it also increased in the latter (Additional file 8 b and c). Therefore, the ratio of Bax/Bcl2, which is a rheostat determining susceptibility to apoptosis [55], in the livers of HFrD-fed *A/A* ^*Hep*^ mice was substantially higher than in those of RD- or HFrD-fed *cont.* mice. Also, a proapoptotic protein Bak was significantly reduced in the HFrD-fed *Cont.* mice but not the HFrD-fed *A/A* ^*Hep*^ mice (Additional file 8 b and c). Collectively, these results suggest an HFrD in eIF2α phosphorylation-deficient *A/A* ^*Hep*^ mice promotes the liver expression of proapoptotic genes (*Casp12*, *Noxa*, *Bak*, and *Bax*), thereby possibly contributing to hepatocyte death.

### Long-term HFrD feeding does not activate the canonical UPR pathway

**Fig. 4:**
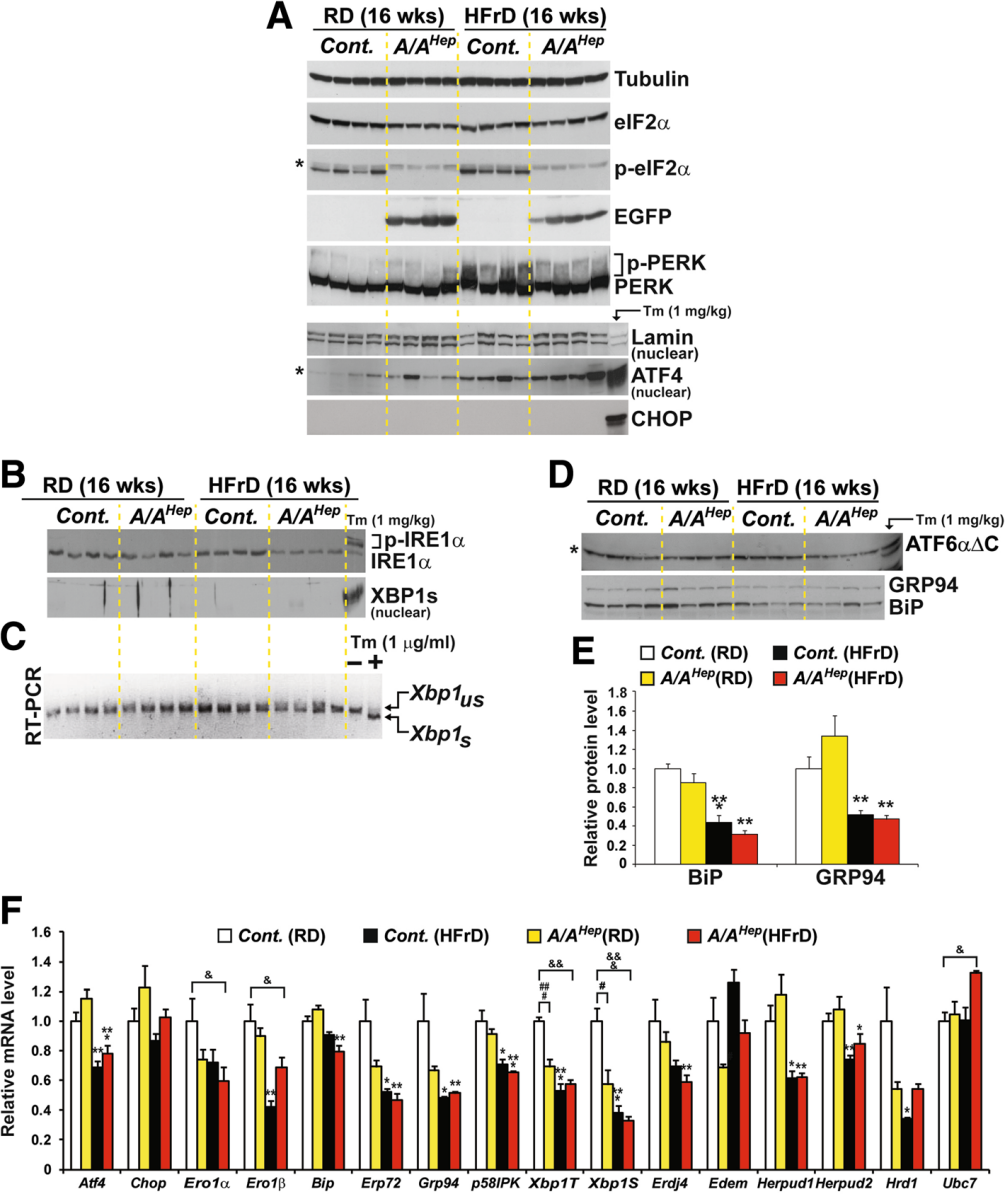
High fructose diet deregulates three UPR pathways. **a** Western blot analysis of PERK pathway proteins in liver lysates from 7-month-old *Cont.* and *A/A* ^*Hep*^ mice fed an RD or an HFrD for 16 wks. Nuclear fractions from liver lysates were used to detect ATF4. The volumes of nuclear fractions used were based on nuclear Lamin A/C protein. The liver tissue of a *control* mouse injected with tunicamycin-(Tm, 1 mg/kg body weight) was used to assess the exact size of ATF4 and CHOP. The * indicates non-specific bands in the Western blot. **b** Western blot analysis of IRE1α and spliced XBP1 (XBP1s), and RT-PCR analysis of *Xbp1* splicing in liver tissues of 7-month-old *Cont.* and *A/A* ^*Hep*^ mice fed an RD or an HFrD for 16 wks. The liver tissue of a *control* mouse injected with Tm was used to assess the exact size of phosphorylated-IRE1α (*last lane in the upper panel*) and spliced XBP1 proteins (*last lane in the lower panel*). The same nuclear fractions in Fig. 4a were used to detect nuclear XBP1s. **c** RT-PCR analysis of *Xbp1* mRNA splicing in liver tissues from 7-month-old *Cont.* and *A/A* ^*Hep*^ mice fed an RD or an HFrD for 16 wks. Total RNA samples were prepared and RT-PCR analysis was performed with a primer set flanking the intron in unspliced *Xbp1* mRNA. PCR products represent unspliced (*Xbp1*_*us*_) and spliced (*Xbp1*_*s*_) species. Total RNA samples from mouse embryonic fibroblasts treated with/without Tm (1 μg/ml) were used to indicate the exact sizes of both *Xbp1*_*us*_ and *Xbp1*_*s*_ species. **d** Western blot analysis of cleaved ATF6α, Grp94 and BiP in liver lysates of 7-month-old *Cont.* and *A/A* ^*Hep*^ mice fed an RD or an HFrD for 16 wks. The liver tissue of a *control* mouse injected with Tm was used to assess the exact size of the N-terminal fragment (ATF6αΔC) of ATF6α (the last lane in the upper panel). The * indicates non-specific bands in the Western blot. **e** Densitometric quantification of BiP and Grp94 in the lower panel of d). Values were normalized against tubulin levels. Data are means ± SEM (*n* = 4 mice per group); **p < 0.01 and ***p < 0.001; RD vs HFrD in the same genotype. **f** Quantitative real-time PCR analysis of the expression of UPR genes in liver tissues from 7-month-old *Cont.* and *A/A* ^*Hep*^ mice fed an RD or an HFrD for 16 wks. Data are means ± SEM (*n* = 5 ~ 6 mice per group); *p < 0.05, **p < 0.01 and ***p < 0.001; RD vs HFrD of the same genotype, ^#^ *p* < 0.05 and ^###^ *p* < 0.001; *Cont.* vs *A/A* ^*Hep*^ *,* ^&^ *p* < 0.05, ^&&^ *p* < 0.01 and ^&&&^ *p* < 0.001; *Cont.*(RD) vs *A/A* ^*Hep*^(HFrD)

Several groups have reported that an HFrD activates the UPR in the liver [11, 12, 51]. Therefore, we checked whether long-term HFrD feeding activated the UPR in the livers of our mice. Western blot analysis of liver lysates from mice fed an HFr diet for 5 or 16 wks revealed persistent activation of PERK in both *Cont.* and *A/A* ^*Hep*^ mice (Fig. 2j and Fig. 4a). Therefore, since phosphorylation of eIF2α was increased in the livers of *Cont.* mice by the HFrD (1.9-fold in Additional file 9 or 1.3-fold in Fig. 4a), whereas eIF2α phosphorylation was not detected in livers of either regular diet (RD)-fed or HFrD-fed *A/A* ^*Hep*^ mice (Fig. 2j, Fig. 4a, and Additional file 9) due to deletion of the *floxed fTg* transgene by CRE recombinase (Additional file 1 a and d), it was reasonable to expect that the PERK/eIF2α downstream target genes ATF4 and CHOP [17, 18] would be absent from the livers of the HFrD-fed *A/A* ^*Hep*^ mice, as was the case (Fig. 4a); however, interestingly, ATF4 and CHOP expression were also not observed in the livers of the HFrD-fed *Cont.* mice, even though they were easily detected in liver tissues of *Cont.* mice exposed to Tm (Fig. 4a). Furthermore, activation of IRE1α (including its downstream target XBP1 [56]) and ATF6α [57] was also not detected in livers of either *Cont.* or *A/A* ^*Hep*^ mice under HFrD conditions (Fig. 4 b–d). In addition, regardless of eIF2α phosphorylation, the HFrD down-regulated expression of the ATF6α downstream target genes (BiP and GRP94) [58, 59] (Fig. 4d and e). Therefore, it seems that long-term high fructose consumption does not activate the canonical UPR pathway in our animal models.

Since eIF2α phosphorylation is required in pancreatic beta cells for optimal expression of many of the downstream targets of the three UPR sensors (IRE1α, PERK, and ATF6α) [29], we investigated how eIF2α phosphorylation in hepatocytes or an HFrD affects expression of the UPR genes. First, on the regular diet *A/A* ^*Hep*^ mice displayed significantly decreased expression of *Xbp1T* and *Xbp1S* and slightly down-regulated expression of *Ero1α*, *Erp72*, *Grp94*, *Edem1* and *Hrd1* (Fig. 4f) compared to *Cont.* mice, suggesting that eIF2α phosphorylation in hepatocytes is required for optimal expression of a subset of UPR genes. Second, to our surprise, the HFrD reduced the expression of many UPR genes (ER chaperones including UPR transcription factors; *Ero1α*, *Ero1β*, *BiP*, *Erp72*, *Grp94*, *p58* ^*IPK*^, *Atf4* and *Xbp1-T/S* and ERAD components; *Erdj4*, *Herpud1*, *Herpud2* and *Hrd1*) in the livers of both *Cont.* and *A/A* ^*Hep*^ mice compared to that of RD-fed *Cont.* mice (Fig. 4f). However, an ultrastructural analysis revealed that the HFr diet, but not the RD, induced significant distention of the ER compartment in hepatocytes of both *Cont.* and *A/A* ^*Hep*^ mice (arrowheads in Additional file 10), indicating that actually fructose-metabolizing hepatocytes of both *Cont.* and *A/A* ^*Hep*^ mice are under ER stress although the expression of UPR genes is dysregulated. Collectively, these data show that long-term HFrD feeding inhibits activation of the UPR sensors (IRE1α and ATF6α) and expression of their downstream genes, whereas it persistently activates PERK/eIF2α phosphorylation-dependent pathways without expression of the proapoptotic *Atf4* and *Chop* genes. Further studies are required to clarify the meaning of this persistent activation of PERK/eIF2α phosphorylation in the livers of HFrD-fed mice. However, under HFr conditions, *A/A* ^*Hep*^ mice are exposed to total dysregulation of the three UPR pathways including PERK/eIF2α phosphorylation-dependent pathway. Consequently, eIF2α phosphorylation deficiency can be an additional risk factor in hepatocytes exposed to metabolic stress (such as ER stress) induced by an excess of a nutrient such as fructose.

### eIF2α phosphorylation is required to maintain NADHP and GSH levels in middle-aged mice

No significant difference was observed in the expression of UPR genes between HFrD-fed *Cont.* mice and HFrD-fed *A/A* ^*Hep*^ mice despite the fact that the high fructose diet suppressed UPR gene expression. Hence the reduced UPR gene expression may not be a major cause of the hepatocyte death in the HFrD-fed *A/A* ^*Hep*^ mice. However, the data in Fig. 1e–g indicate that eIF2α phosphorylation-deficient hepatocytes are actually very susceptible to oxidative stress. Therefore, we next focused on the cytotoxic effect of ROS, which can be accumulated during fructose metabolism [3].

**Fig. 5:**
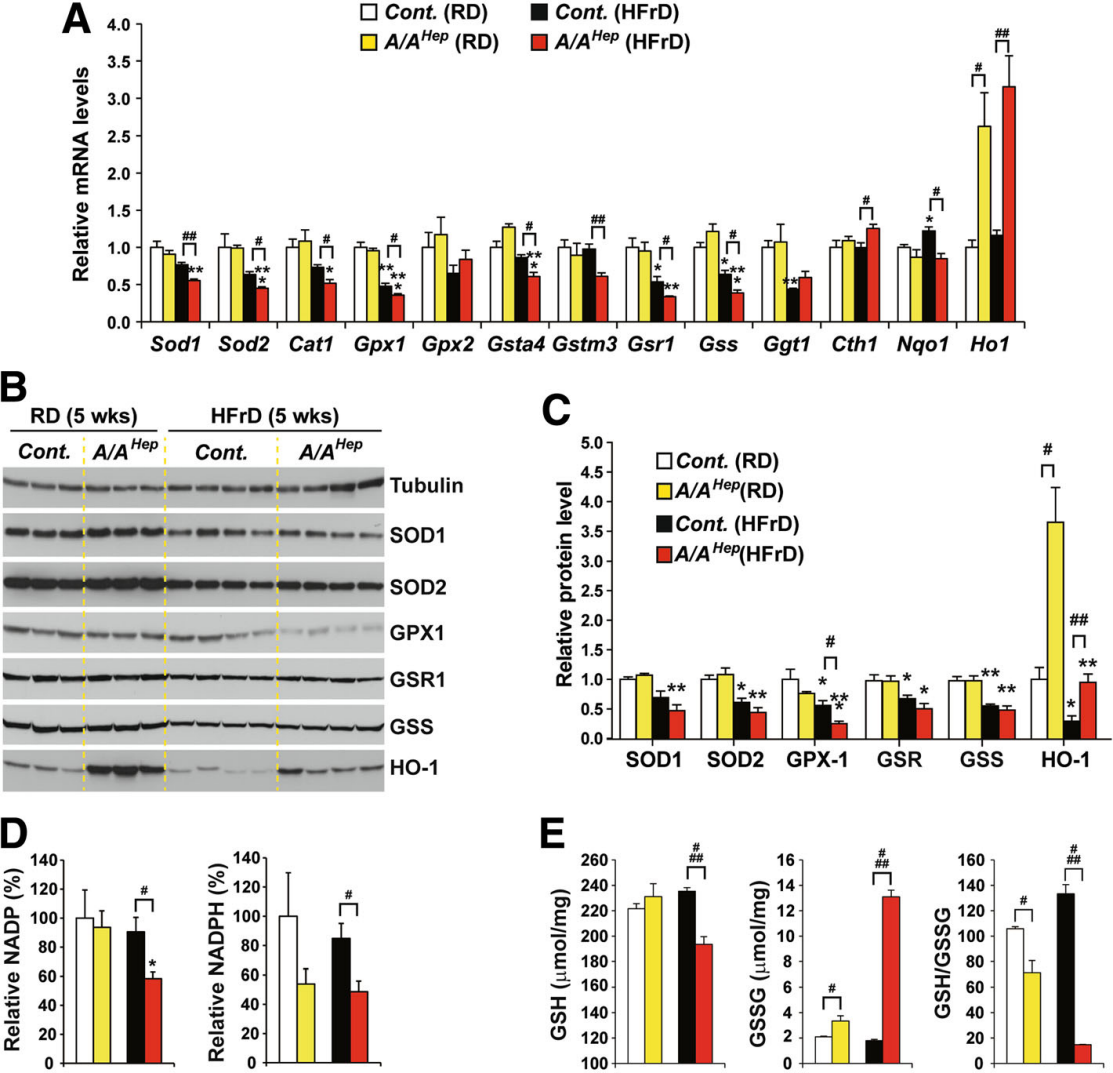
A high fructose diet lowers NADPH and GSH levels required for scavenging of ROS in liver tissues of *A/A* ^*Hep*^ middle-aged mice. **a** Quantitative real-time PCR analysis of the expression of selected mRNAs (ROS-defense genes) in liver tissues from 13-month-old *Cont.* and *A/A* ^*Hep*^ mice fed an RD or an HFrD for 5 wks. Data are means ± SEM (*n* = 4 ~ 5 mice per group); *p < 0.05, **p < 0.01 and ***p < 0.001; RD vs HFrD in the same genotype, ^#^ *p* < 0.05 and ^##^ *p* < 0.01; *Cont.* vs *A/A* ^*Hep*^. **b** Western blot analysis of antioxidant proteins in liver lysates from 13-month-old *Cont.* and *A/A* ^*Hep*^ mice fed an RD or an HFrD for 5 wks. **c** Densitometric quantification of protein expression levels in (b). Values were normalized against tubulin levels. Data are means ± SEM (*n* = 3 or 4 mice per group); ^#^ *p* < 0.05 and ^##^ *p* < 0.01; *Cont.* vs *A/A* ^*Hep*^ and *p < 0.05, **p < 0.01, and ***p < 0.001; RD vs HFrD in the same genotype. **d** Quantification of NADP and NADPH levels in liver tissues of animals fed an RD or an HFrD for 5 wks. Data are means ± SEM (*n* = 4 ~ 5 mice per group); *p < 0.05; RD vs HFrD, ^#^ *p* < 0.05; *Cont.* vs *A/A* ^*Hep*^. **e** Quantification of GSH and GSSG levels in liver tissues of animals fed an RD or an HFrD for 5 wks. Data are means ± SEM (*n* = 4 ~ 5 mice per group); ^#^ *p* < 0.05 and ^###^ *p* < 0.001; *Cont.* vs *A/A* ^*Hep*^

To assess whether the HFrD treatment affects the expression of ROS-related genes in the hepatocytes of adult and middle-aged *A/A* ^*Hep*^ mice, we measured the mRNAs levels of superoxide (*Sod1* and *Sod2*)- and hydrogen peroxide (*Cat1*, *Gpx1*, and *Gpx2*)-scavenging enzymes, as well as glutathione (GSH) S-transferases (*Gsta4* and *Gstm3*), glutathione-disulfide reductase (*Gsr1*), GSH biogenesis-related enzymes (*Gss and Cth1*), and NADPH-dependent antioxidant enzymes (*Nqo1* and *Ho-1).* With an RD, transcript levels of all the tested genes except *Ho-1* did not differ between *cont.* and *A/A* ^*Hep*^ mice (Fig. 5a and Additional file 11 a), whereas after exposure to the HFr diet for 5 and 16 wks, transcripts of many of the genes were reduced in the livers of the *Cont.* mice (Fig. 5a and Additional file 11 a); in addition, the absence of eIF2α phosphorylation in 13-month-old *A/A* ^*Hep*^ mice further reduced expression of many of these enzymes (Fig. 5a) but not in the *adult A/A* ^*Hep*^ mice (Additional file 11 a). The HFr diets also led to a reduction of these proteins in both adult and 13-month-old animals, and further repressed the level of GPX1, a GSH-utilizing hydrogen peroxide-scavenging enzyme, in 13-month-old *A/A* ^*Hep*^ mice (Fig. 5b and c) but not in adult *A/A* ^*Hep*^ mice (Additional file 11 b and c). It is interesting that the transcript (Fig. 5a and Additional file 11 a) and protein (Fig. 5b and c and Additional file 11 b and c) levels of the antioxidant *Ho-1* gene [60, 61] in *A/A* ^*Hep*^ mice were substantially high than those of the *cont.* mice, regardless of diet, although the HO-1 level in 13-month-old *A/A* ^*Hep*^ mice was lower on the HFr diet (Fig. 5b and c). Thus, the HFr diet reduces the expression of antioxidant enzymes in hepatocytes, and that eIF2α phosphorylation-deficiency plus aging may lead to additional dysregulation of ROS-defense gene expression.

Because the antioxidant enzymes CAT1, GSR1, NQO1, and HO-1 use the reducing power of the cofactor NADPH to maintain their enzymatic activity [62, 63], we measured levels of NADP and NADPH in liver tissues of 13-month-old mice (Fig. 5d). The HFr diet significantly decreased NADP in the *A/A* ^*Hep*^ livers. Furthermore, NADPH levels were lower in both RD-fed and HFrD-fed *A/A* ^*Hep*^ liver tissues than in the corresponding *Cont.* livers (Fig. 5d), suggesting that eIF2α phosphorylation may be important for maintaining NADPH and NADP levels in middle-aged mice. Hence, it is possible that the reduced NADPH level affects the activities of NADPH-dependent antioxidant enzymes (CAT1, NQO1, and HO-1) and this may reduce the antioxidant capacity of the hepatocytes of middle-aged *A/A* ^*Hep*^ mice. Furthermore, the decrease in NADPH level may lead to a decline in GSH because NADPH-dependent GSR1 regenerates reduced GSH from oxidized GSH (GSSG) [62, 63]. Therefore, we measured levels of reduced glutathione (GSH) and oxidized glutathione (GSSG) in liver tissues (Fig. 5e). GSH was unchanged but GSSG was slightly increased in the eIF2α phosphorylation-deficient liver tissues of mice on regular diets (Fig. 5e). However, the HFr diet significantly reduced GSH and increased GSSG in *A/A* ^*Hep*^ livers. As a result, the GSH/GSSG ratio in the liver tissues of *A/A* ^*Hep*^ 13-month-old mice was slightly reduced on an RD and substantially reduced on a HFrD (Fig. 5e). However, the GSH level in the latter was normal after 16 wks although the GSSG level was slightly elevated (Additional file 11 d). These results suggest that eIF2α phosphorylation is required to maintain constant GSH and GSSG levels in middle-aged mice especially during HFrD feeding. Therefore, it is possible that under the HFr conditions, the decline in reduced GSH affects the activities of GSH-dependent antioxidant enzymes (GPX1, GPX2, GSTm4, and GSTa3) [64] and thus reduces hepatocyte antioxidant capacity in middle-aged *A/A* ^*Hep*^ mice. Taken together, these observations suggest that under HFr conditions, eIF2α phosphorylation-deficient hepatocytes in middle-aged mice have reduced expression of antioxidant enzymes (GPX1 and HO-1) and lowered levels of NADPH and GSH, which will lead to a significant decline in cellular antioxidant capacity. This in turn could cause ROS-mediated damage and eventually hepatocyte death in the *A/A* ^*Hep*^ mice.

### HFrD feeding causes ROS-mediated damage in *A/A*^*Hep*^ mutant mice

**Fig. 6:**
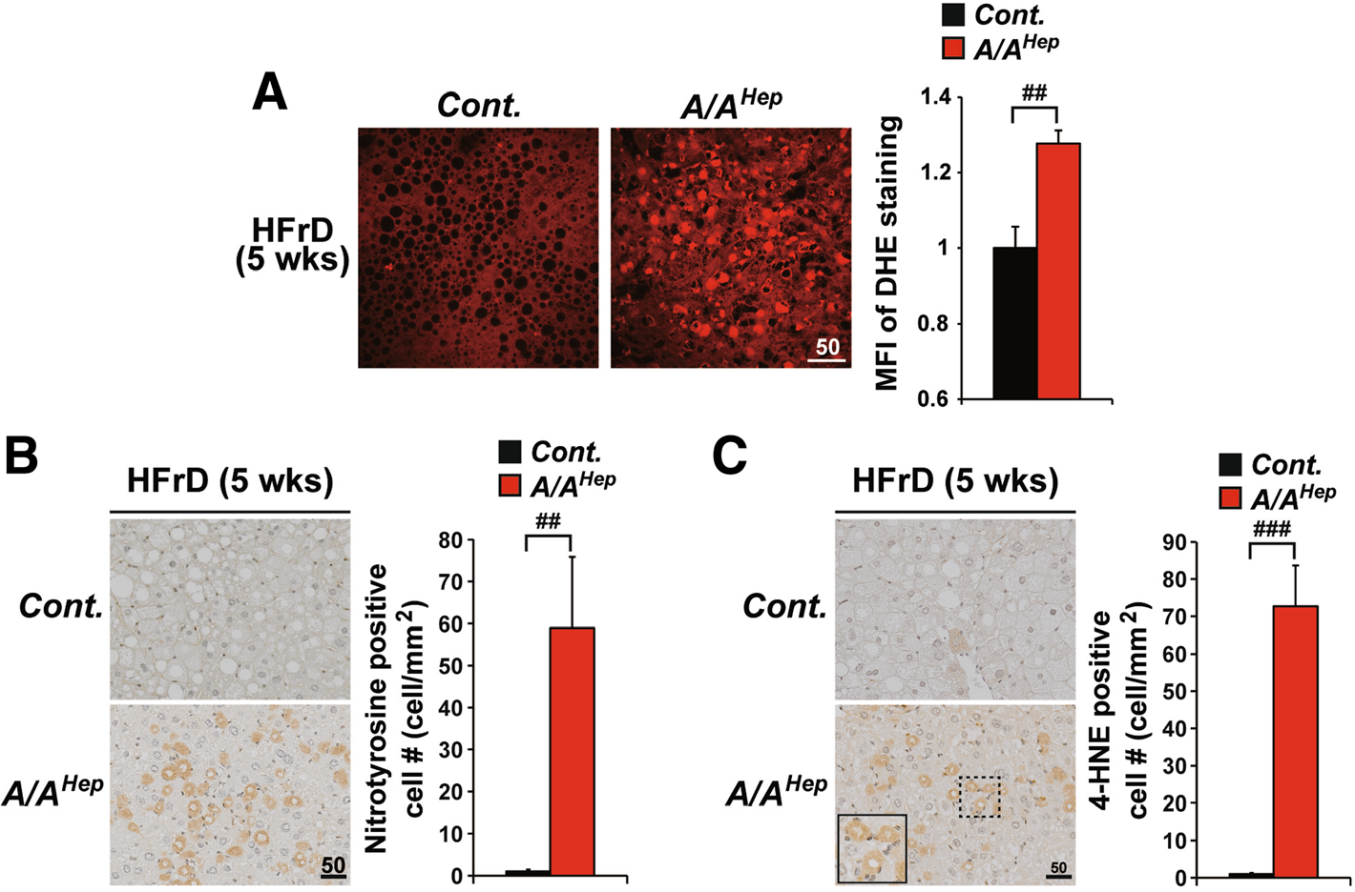
eIF2α phosphorylation is required to prevent ROS accumulation and oxidative damage in liver tissues of HFrD-fed mice. **a** Fluorescence microscope observation of frozen liver sections stained with dihydroethidium (DHE) from 13-month-old *Cont.* and *A/A* ^*Hep*^ mice fed an HFrD for 5 wks, reflecting ROS levels. Representative images are shown (*n* = 3 mice per group). Mean fluorescence intensities (MFI) of DHE staining were measured with image analysis software. Data are means ± SEM (*n* = 3 mice per group; ^##^ *p* < 0.01; *Cont.* vs *A/A* ^*Hep*^. **b** and (**c**) Immunohistochemistry of nitrotyrosine (**b**) and 4-HNE (**c**) in liver sections from 13-month-old *Cont.* and *A/A* ^*Hep*^ mice fed an HFrD for 5 wks, respectively. Representative images are shown (*n* = 4 ~ 5 mice per group). Inset shows a magnified view of the area outlined in the black dotted box. Quantification of nitrotyrosine and 4-HNE-positive cells is shown in the graphs. Data are means ± SEM (*n* = 4 ~ 5 mice per group); ^##^ *p* < 0.01 and ^###^ *p* < 0.001; *Cont.* vs *A/A* ^*Hep*^

We next investigated whether HFrD feeding of *A/A* ^*Hep*^ mice, which leads to a decline in antioxidant capacity, induces ROS accumulation and ROS-mediated tissue damage. Examination of liver sections with a dihydroethidium (DHE) fluorescence microscope revealed weak fluorescence with no visible nuclear fluorescence in the livers of RD-fed control and mutant mice (Additional file 11 e, upper panels), indicating that an RD does not induce ROS accumulation in *A/A* ^*Hep*^ liver tissues. In contrast, under HFrD conditions, DHE fluorescence intensity significantly increased and many DHE-stained nuclei were clearly visible in liver sections of the HFrD-fed 13-month-old (Fig. 6a, middle panel) and adult (Additional file 11 e, lower right panel) *A/A* ^*Hep*^ mice, but not in those of HFrD-fed 13-month-old (Fig. 6a, left panel) and adult (Additional file 11 e, lower left panel) *Cont.* mice. This shows that eIF2α phosphorylation-deficiency can lead to ROS accumulation in fructose-metabolizing hepatocytes.

Next, we analyzed HFrD-mediated oxidative damage in the livers of control and mutant mice. Cellular protein damage due to oxidative stress can occur when superoxide reacts with nitric oxide, leading to peroxynitrite modification of tyrosine residues. Numerous nitrotyrosine-positive hepatocytes were observed in livers of *A/A* ^*Hep*^ mice fed an HFrD for 5 and 16 wks but were very rare in the control mice (Fig. 6b and Additional file 11 f). 4-Hydroxynonenal (4-HNE) is a lipid peroxidation product, and consistent with nitrotyrosine staining results, the number of 4-HNE-positive hepatocytes was greatly elevated in the livers of HFrD-fed *A/A* ^*Hep*^ mice compared to HFrD-fed *cont.* mice (Fig. 6c and Additional file 11 g). Taken together, our results indicate that hepatocyte eIF2α phosphorylation prevents ROS accumulation and ROS-mediated oxidative stress induced by fructose metabolism, and thereby prevents hepatocyte death.

## Discussion

The present study shows that eIF2α phosphorylation in the livers of HFrD-fed middle-aged mice is critical for maintaining antioxidant capacity and thereby mitigating fructose diet-mediated toxicity, whereas it is dispensable for survival of adult mice and contributes minimally to hepatocyte viability in the absence of metabolic stress. When middle-aged mice consume an HFr diet, the absence of hepatocyte eIF2α phosphorylation results in decreased expression of several ROS-defense genes and lower levels of NADPH and GSH, cofactors of the ROS-defense enzymes. This leads to the accumulation of ROS and oxidative damage, and the progression of NASH.

Several reports including ours have highlighted the physiological importance of eIF2α phosphorylation in multiple cell types and tissues, such as pancreatic beta cells [25, 29], intestinal epithelial cell [65] and hepatocytes [25, 66], as well as the hippocampus [67], in a variety of mouse models of eIF2α phosphorylation deficiency. eIF2α phosphorylation was shown to be essential for optimal expression of many UPR genes and the maintenance of cellular homeostasis and normal function in target cells [25, 65–67]. However, the effect eIF2α phosphorylation deficiency on cell viability varied from autonomous cell death [25, 29] to increased susceptibility to infection and inflammatory insults [65]. These reports suggest that different effects on cell viability in different eIF2α phosphorylation-deficient cell types may be due to different cellular contexts and environments. In line with this idea, we observed that hepatic eIF2α phosphorylation deficiency itself induced spontaneous death of few hepatocytes, but particularly the HFrD consumption plus aging including long-term eIF2α phosphorylation deficiency further augmented hepatocellular death via apoptosis and necroinflammation in eIF2α phosphorylation-deficient hepatocytes..

Numerous studies with humans and rodent models indicate that fructose, a highly lipogenic sugar, has profound metabolic effects on the liver [5, 7] and its consumption is associated with several detrimental components of metabolic syndrome, including dyslipidemia, steatosis, inflammation, impaired glucose homeostasis, insulin resistance and hypertension [3, 5, 7, 8]. In addition, fructose-enriched diets can induced ER stress [11, 13, 68], mitochondrial dysfunction [3, 10], and oxidative stress [3, 9], which can affect cell viability. However, there are not many reports that fructose-enriched diets can cause hepatocyte death or hepatic injury in in vivo animal models [69].

Unexpectedly we observed in the present study that a long-term high fructose diet did not induce the canonical UPR pathways [11, 12, 51]. On the contrary, it led to failure to activate the IRE1α and ATF6α pathways and decreased expression of several UPR genes, although it induced phosphorylation of PERK/eIF2α and caused expansion of the ER. However we found that IRE1α and PERK/eIF2α were phosphorylated after just 3 days of the HFrD (data not shown) as shown by other groups [11, 13, 68] but that activation of the IRE1α proteins did not persist (Fig. 4a). Therefore, we conclude that in our mouse model, high fructose may activate UPR via ER stress at early times but that prolonged fructose metabolism dysregulates ER functions, leading to reduced expression of UPR genes by inhibiting the IRE1α and ATF6α pathways and non-canonically activating the PERK/eIF2α pathway. Ultimately, both wild type and eIF2α phosphorylation-deficient hepatocytes will be exposed to unresolved ER stress. We do not know why HFrD-mediated UPR pathway activation differs in different animal models, but different mouse strains (C57BL/6 (BL6) mice vs BALB/c mice) display divergent ER stress response to fructose feeding [70]. As described in Methods, our mouse model has a mixed genetic background. Therefore, it is possible that this mixed background contributes to the distinctive pattern of UPR pathway activation in response to the HFr diet. However, further studies are required to establish the molecular basis of the differences and how prolonged fructose metabolism dysregulates the UPR pathway.

Several groups have reported that a fructose-rich diet can alter antioxidant status, cause ROS accumulation, and induce ROS-mediated damage in the hepatocytes and livers of rodent models [9, 38, 71]. Little is known about how fructose metabolism produces ROS. It is believed that the linear ketal form of fructose can be conjugated to the ε-amino group of lysine in proteins by a non-enzymatic reaction, termed the Maillard reaction [72]. During this non-enzymatic fructosylation, each event releases a superoxide radical [3, 73]. In addition, a fructose-enriched diet reduces the expression and activity of several antioxidant enzymes (such as SODs1/2, Catalase, and GPX1) in the livers of rats [37, 38, 71] through an unknown mechanism, and the deterioration of cellular antioxidant status will increase the accumulation of ROS induced by fructose metabolism. In the present study, we found that regardless of eIF2α phosphorylation ability, the HFr diet also decreased the mRNA and protein levels of several ROS-defense genes (Fig. 5a–c and Additional file 11 a–c). These changes consequently lowered resistance to oxidative stress. In addition, HFrD feeding for 5 wks further suppressed the expression of their mRNAs in the livers of eIF2α phosphorylation-deficient *A/A* ^*Hep*^ middle-aged mice (Fig. 5a). Moreover, the expression of the hydrogen peroxide scavenger enzyme GPX1 protein was diminished in HFrD-fed *A/A* ^*Hep*^ mice, compared to HFrD-fed *cont.* mice (Fig. 5b and c). It has also been reported that *Gpx1*- null mice have increased susceptibility to oxidative stress [74]. Therefore, we suggest that all these changes in expression of antioxidant genes due to the HFr diet in the eIF2α phosphorylation-deficient hepatocytes of middle-aged mice erode antioxidant capacity.

In addition, the significant increase of HO-1, a potent endogenous antioxidant enzyme in the livers of RD-fed *A/A* ^*Hep*^ mice can be interpreted as showing that eIF2α phosphorylation deficiency weakens the antioxidant status of hepatocytes, so that increased amounts of HO-1 are required to restore cellular antioxidant status. However, interestingly, 5 wks HFrD feeding of both *cont.* and *A/A* ^*Hep*^ middle-aged mice suppressed the level of HO-1 protein without changing its mRNA level (Fig. 5a–c), whereas 16 wks of HFrD feeding of adult *A/A* ^*Hep*^ mice instead increased HO-1 without changing its mRNA level (Additional file 11 a–c). These effects of fructose on HO-1 can thus weaken the antioxidant status of eIF2α phosphorylation-deficient hepatocytes in middle-aged mice or conversely potentiate it in adult mice. However, further studies are required to unravel the many unanswered questions about the alterations of HO-1 by eIF2α phosphorylation and changing diets.

Glutathione (GSH) is the most abundant thiol-containing component of living cells and is believed to play an important role as an antioxidant. Its biosynthesis and regeneration occur in the cytosol in a tightly regulated manner in which the availability of its amino acid components (e.g. cysteine) and many enzymes (e.g. glutamate cysteine ligase (GCL), GSH synthetase (GSS) and GSH reductase1 (GSR1)) are involved [75]. In particular, glutathione depletion is a common feature of apoptotic cell death triggered by a wide variety of stimuli including activation of death receptors, stress, environmental agents, and cytotoxic drugs [76]. As already seen by others [37, 38, 71], excessive dietary fructose consumption reduced the mRNA and protein levels of genes involved in GSH biosynthesis (GSS and GGT1) and regeneration (GSR1) in the livers of HFrD-fed middle-aged (Fig. 5a–c) and adult (Additional file 11 a–c) mice. However, there was no difference in the levels of those proteins between *control* and *A/A* ^*Hep*^ mice in HFrD conditions. Instead we observed that eIF2α phosphorylation deficiency in middle-aged mice downregulated NADPH, and reduced GSH in the HFrD condition (Fig. 5d and e). However, these changes were not observed in the livers of *A/A* ^*Hep*^ mice fed an HFrD for 16 wks. Because NADPH is a cofactor of several antioxidant enzymes such as CAT1, GSR1, NQO1, HO-1, BVR (biliverdin reductase) and TrxR (thioredoxin reductase) [62], a decline of its level will decrease these enzymatic activities without any change in their protein levels, and reduce hepatic antioxidant capacity. In addition, the lower amount of NADPH together with a decrease in GSR1 might be expected to contribute to a fall in reduced GSH regenerated from oxidized GSH (GSSG) in the livers of HFrD-fed *A/A* ^*Hep*^ middle-aged mice. Consistent with this prediction, we observed reduced GSH levels in the livers of HFrD-fed *A/A* ^*Hep*^ middle-aged mice (Fig. 5e). Moreover, the simultaneous decrease in both HO-1 (Fig. 5b and c) and NADPH (Fig. 5d) levels in the HFrD condition will further worsen antioxidant capacity in eIF2α phosphorylation-deficient hepatocytes. However, several questions are unanswered such as what causes the downregulation of mRNA and protein levels of GSH biosynthesis and regeneration-related genes, how eIF2α phosphorylation modulates levels of NADP and NADPH, and why NADPH and GSH levels are only reduced in the livers of middle-aged *A/A* ^*Hep*^ mice.

In addition to the decline in HFr diet-mediated hepatic antioxidant capacity in *A/A* ^*Hep*^ middle-aged mice, O’Brien’s group have suggested that there is a marked increase in fructose susceptibility if the hepatocytes are exposed to H_2_O_2_, a product generated by the NADPH oxidase of activated immune cells. They reported that fructose-induced hepatocyte toxicity was increased approximately 100-fold by nontoxic levels of H_2_O_2_ [46, 47]. Similar to their experimental conditions, we observed that expression of NADPH oxidase (*Nox2*) and its component genes (*Phox22*, *40,* and *67*) was elevated in the livers of HFrD-fed *A/A* ^*Hep*^ mice (Figs. 2h and 3i). Consistent with this gene expression result, we found that most 4-HNE-positive hepatocytes were surrounded by infiltrated immune cells in the livers of HFrD-fed *A/A* ^*Hep*^ middle-aged and adult mice (Fig. 6c and Additional file 11 g). Therefore, we propose that the marked increase of fructose susceptibility may be related to exposure of eIF2α phosphorylation-deficient hepatocytes to H_2_O_2_ derived from immune cells recruited and activated under the HFrD conditions.

In what concerns the development of liver fibrosis in HFrD-fed *A/A* ^*Hep*^ mutant mice, our results present several lines of evidence that it is a result of necroinflammation, which is an autoamplification loop driven by regulated necrosis and inflammation [77, 78]. For necrosis, microscopic observation revealed that there was substantially increased necrotic cell death in the liver tissues of *A/A* ^*Hep*^ mutant mice under the conditions of HFr diet and/or aging although more works are required to confirm the cell death mode (Fig. 2a, b, Fig. 3b, and Additional file 4a and Additional file 6 a–c). Interestingly there is a big difference between necrotic cell number and cleaved cas-3 positive cell number in the liver tissues of *A/A* ^*Hep*^ mutant mice (compare Fig. 2b with Fig. 2e and Fig. 3b with Fig. 3d). A higher number of necrotic cells suggest that necrotic cell death plays a more significant role than apoptosis in mediating the loss of hepatocytes in *A/A* ^*Hep*^ mutant mice. Nonetheless, there are significant levels of apoptosis (Figs. 2e and 3d and Additional file 4c and Additional file 6f) in the liver tissues of *A/A* ^*Hep*^ mutant mice too. Therefore, we have to determine the relative significance of necrosis and apoptosis in the hepatocellular death of *A/A* ^*Hep*^ mutant mice. For inflammation in the liver tissues of *A/A* ^*Hep*^ mutant mice having necrotic hepatocellular death, there are several evidences, such as increased infiltrated immune cells (possibly Kupffer cells and neutrophils) around dying or dead hepatocytes (Figs. 2a and 6c and Additional file 6a, b, c and Additional file 11 g) and increased expression of proinflammatory cytokine genes (*Il6, Tnfα* and *Il1β*) (Figs. 2g and 3h) and ROS-generating NADPH oxidase genes (*Nox2*, *Phox22*, *Phox40, Phox47* and *Phox67*) (Figs. 2h and 3i) produced from recruited and activated immune effector cells (such as Kupffer cells and neutrophils). Therefore, livers of *A/A* ^*Hep*^ mutant mice under the conditions of HFr diet and/or aging will experience the necroinflammatory auto-amplification loop of necrotic cell death and inflammation. Under the necroinflammation, factors (such as *Tgf-β1, Il6, Tnfα* and *Il1β*) released by the inflammatory cells lead to transformation of hepatic stellate cells into a myofibroblast-like phenotype [79], which play crucial roles in hepatic fibrogenesis [48–50]. Therefore, we observed increased expression of several proinflammatory cytokine genes (*Tgf-β1, Il6, Tnfα* and *Il1β* in Figs. 2g and 3h) and liver fibrosis marker genes (*Col1A1*, *Col3A1*, *Timp1* and *Pdgfr-β* in Figs. 2g and 3h) in the livers of the HFrD-fed *A/A* ^*Hep*^ mice, suggesting that the development of liver fibrosis in our animal model can be a result of necroinflammation.

In summary, under HFr conditions, all hepatocytes may already have a lowered antioxidant capacity because of fundamentally dysregulated UPR and ROS-defense gene expression. Also, the eIF2α phosphorylation-deficient hepatocytes in middle-aged mice will be exposed to the following multifactorial events: first, the decrease in CAT1, NQO1, HO-1 and especially GSR1 activities due to the fall in NADPH will reduce antioxidant capacity and contribute to a decline in reduced GSH; next, the decline in GSH needed for the activities of GPX1, GPX2, GSTa4 and GSTm3 will further reduce antioxidant capacity, and the diminished amount of GPX1 protein will exacerbate oxidative stress. All these harmful events together will destroy the antioxidant capacity of eIF2α phosphorylation-deficient hepatocytes, cause ROS accumulation and damage, and eventually kill the hepatocytes of middle-aged mice. Furthermore, damage to hepatocytes may result in the recruitment and stimulation of H_2_O_2_-producing NOX2-expressing inflammatory cells, and in this way fructose-induced hepatocytes will be caught in a vicious circle of hepatocyte death and leukocyte infiltration. However, these multifactorial events, except for the recruitment of NOX2-expressing inflammatory cells, were not observed in the eIF2α phosphorylation-deficient hepatocytes of adult mice on an HFrD, but only in those of middle-aged mice, indicating that the fructose diet-mediated toxicity may be aggravated by detrimental factors accumulated during the aging process and by long-term eIF2α phosphorylation deficiency.

## Conclusions

Regardless of genetic background, long-term feeding of mice with an HFr diet induces dyslipidemia and hepatic steatosis, causes dysregulation of UPR sensor activation and UPR gene expression, and leads to decreased expression of several ROS-defense genes. Nonetheless these changes are not enough to induce hepatocyte death. However, if they are combined with eIF2α phosphorylation-deficiency and aging, the adverse effects of the diet on antioxidant capacity, inflammation, and cell viability are reinforced. These effects include reduced expressions of GPX1 and HO-1, lowered levels of NADPH and GSH, and increased infiltration of leukocytes. Our findings provide novel insights into the physiological roles of eIF2α phosphorylation in preventing diet-induced metabolic stress and aging.

## Additional files

Additional file 1: eIF2α phosphorylation in hepatocytes is dispensable for survival of adult mice. (a) Diagram depicting the four genotypes of mice used in these experiments. *S/A* and *A/A* represent heterozygous and homozygous *eIF2α* Ser51Ala (*) mutation(s) in exon 2 of one *eIF2α* allele and both *eIF2α* alleles, respectively. *fTg/0* represents the floxed wild type (WT) *eIF2α* transgene driven by the CMV enhancer and chicken β-actin promoter (Enh-Pro). *The loxP* sequences (black arrowheads) allow excision of the WT *eIF2α* floxed transgene (*fTg*) and expression of EGFP, an indicator of recombination. *CRE* ^*Hep*^ */0* represents the Cre recombinase transgene driven by the promoter (Alfp) of *Alb1* (encoding albumin) and the enhancer of *Afp* (encoding alpha-fetoprotein). (b) Efficiency of deletion of the *fTg* in liver tissues. Results from quantitative RT-PCR analyses of transgenic and total *eIF2α* mRNAs are shown. Data are means ± SEM (*n* = 4 ~ 5 mice per group); ^###^ *p* < 0.001; *Cont.* vs *A/A* ^*Hep*^. (c) Western blot analysis of eIF2α protein expression driven by the *fTg* in liver tissues. To quantity expression of eIF2α, blots were incubated with anti-eIF2α antibody followed by IRDye-800 goat anti-rabbit IgG (LI-COR). Membranes were scanned on an Odyssey scanner (LI-COR) (lower two panels in left panels) and quantified with the Odyssey Software package. (d) Western blot analysis of liver lysates in *Cont.* and *A/A* ^*Hep*^ mice at the indicated times after Tm injection. *Cont.* mice and *A/A* ^*Liv*^ mice were injected with vehicle or tunicamycin (Tm, 1 mg/kg). (e) Body weight measurements of *fTg* -deleted *A/A* ^*Liv*^ mice. At the weeks, body weight was measured in both male and female mice. Data are means ± SEM (*n* = 6-14 mice per group). (PDF 1987 kb)

Additional file 2: Table S2. PCR primers. (XLS 40 kb)

Additional file 3: Table S1. Survival ratio of young progeny (2 ~ 3 month old) from breeding of *A/A;fTg/fTg* X *S/A;CRE* ^*Hep*^ */0* mice. (XLSX 10 kb)

Additional file 4: Hepatocyte death and fibrosis in 13 month-old *A/A* ^*Hep*^ mice fed a regular diet (RD) or a 60% high fructose diet (HFrD) for 5 wks (a) Hematoxylin and eosin (H&E)-stained images of liver tissue sections from 13-month-old *Cont.* (*A/A-fTg*) (*n* = 5) and *A/A* ^*Hep*^ mice (*n* = 8) fed an RD. Representative images are shown. (b) TUNEL and (c) Cleaved caspase-3-stained images of liver tissue sections from 13-month-old *Cont.* (*A/A-fTg or S/A-fTg*) and *A/A* ^*Hep*^ mice fed an RD (*n* = 5 ~ 8 mice per group) or an HFrD (*n* = 7 mice per group) for 5 wks. The arrowheads indicate positive cells. Representative images are shown. (d) Sirius red-stained and (e) Masson’s trichrome-stained images of liver tissue sections from 13-month-old *Cont.* (*A/A-fTg*) (*n* = 5) and *A/A* ^*Hep*^ mice (*n* = 8) fed an RD. Representative images are shown. (PDF 5270 kb)

Additional file 5: Comparison of hepatic and serum triglyceride levels in RD and HFrD-fed mice. (a) Weight gain of *Cont.* and *A/A* ^*Hep*^ mice on an RD or an HFrD for 13 wks. Data are means ± SEM (*n* = 6 mice per group); *p < 0.05 and **p < 0.01; RD vs HFrD in the same genotype. (b) Hepatic and (c) serum triglyceride levels of *Cont.* and *A/A* ^*Liv*^ mice fed an RD or an HFrD for 16 wks. Data are means ± SEM (*n* = 6 mice per group); *p < 0.05; RD vs HFrD. (d) Quantitative real-time PCR analysis of expression of selected genes (fatty acid synthesis and metabolism) in liver lysates from *Cont.* and *A/A* ^*Hep*^ mice fed an RD or an HFrD for 16 wks. Data are means ± SEM (*n* = 6 mice per group); *p < 0.05, **p < 0.01 and ***p < 0.001; RD vs HFrD. (PDF 929 kb)

Additional file 6: eIF2α phosphorylation in adult mice prevents hepatocyte death induced by a long-term HFrD. (a) Hematoxylin and eosin (H&E)-stained images of paraffin-embedded liver sections from 7-month-old *Cont.* and *A/A* ^*Hep*^ mice fed an RD or an HFrD for 16 wks. Inset shows a magnified view of the area outlined in the black dotted box. The image reveals a necrotic hepatocyte surrounded by leukocytes. (b) Hematoxylin and eosin (H&E)-stained images of liver tissue sections from 7-month-old *A/A* ^*Liv*^ mice fed an HFrD for 16 wks. Dashed lines delineate the area consisting of ruptured dead cells (asterisks) and dying hepatocytes with fragmented nuclei (red arrow) and atypic nuclei (arrow head). (c) Hematoxylin and eosin (H&E)-stained images of OCT-embedded liver sections from 7-month-old *Cont.* and *A/A* ^*Hep*^ mice fed an RD or an HFrD for 16 wks. To prepare frozen blocks, tissue was embedded in Tissue-Tek OCT compound (Sakura Finetek). OCT-embedded sections (7 μm) of frozen livers were stained with haematoxylin and eosin (H&E). Inset shows a magnified view of the area outlined in the black dotted box. The image reveals a necrotic hepatocyte surrounded by leukocytes. (d) Masson’s trichrome-stained images of liver tissue sections from 7-month-old *Cont.* and *A/A* ^*Hep*^ mice fed an RD for 16 wks. Representative images are shown (*n* = 6 mice per group). (e) TUNEL analysis and (f) Immunohistochemical staining of cleaved caspase3 of liver tissue sections from 7-month-old *Cont.* and *A/A* ^*Hep*^ mice fed an RD or an HFrD for 16 wks. The arrowheads indicate positive cells. Representative images are shown (*n* = 6 mice per group). (PDF 6082 kb)

Additional file 7: The severity of HFrD-mediated damage is higher in middle-aged *A/A* ^*Hep*^ mice. (a) The data (serum ALT level, necrotic cell #, TUNEL positive cell #, and cleaved caspase 3 positive cell # of RD-fed adult *A/A* ^*Hep*^ mice from Fig. 3a–d and RD-fed middle-aged *A/A* ^*Hep*^ mice from Fig. 2b–e) were reused to generate the graphs. The data were statistically analyzed to evaluate the difference of measured values. Data are means ± SEM (*n* = 6 mice for RD-fed adult *A/A* ^*Hep*^ mice and *n* = 8 mice for RD-fed middle-aged *A/A* ^*Hep*^ mice). ns stands for no significant. (b) The data (serum ALT level, necrotic cell #, TUNEL positive cell #, and cleaved caspase 3 positive cell # of 16 wks HFrD-fed adult *A/A* ^*Hep*^ mice from Fig. 3a–d and 5 wks HFrD-fed middle-aged *A/A* ^*Hep*^ mice from Fig. 2b–e) were reused to generate the graphs. The data were statistically analyzed to evaluate the difference of measured values. Data are means ± SEM (*n* = 6 mice for 16 wks HFrD-fed adult *A/A* ^*Hep*^ mice and *n* = 7 mice for 5 wks HFrD-fed middle-aged *A/A* ^*Hep*^ mice); ^#^ *p* < 0.05 and ^##^ *p* < 0.01; Adult vs Middle-aged. ns stands for no significant. (PDF 1137 kb)

Additional file 8: A high fructose diet increases expression of proapoptotic genes in liver tissues of *A/A* ^*Hep*^ mice. (a) Quantitative real-time PCR analysis to assess the expression of selected genes (anti/proapoptotic genes) in liver tissues from 7-month-old *Cont.* and *A/A* ^*Hep*^ mice fed an RD or an HFrD for 16 wks. Data are means ± SEM (*n* = 5 ~ 6 mice per group); *p < 0.05 and **p < 0.01; RD vs HFrD in the same genotype, ^#^ *p* < 0.05, ^##^ *p* < 0.01 and ^###^ *p* < 0.001; *Cont.* vs *A/A* ^*Hep*^ *,* ^&^ *p* < 0.05 and ^&&&^ *p* < 0.001; *Cont.*(RD) vs *A/A* ^*Hep*^(HFrD). (b) Western blot analysis of selected anti/proapoptotic proteins in liver lysates from 7-month-old *Cont.* and *A/A* ^*Hep*^ mice fed an RD or an HFrD for 16 wks. (c) and (d) Densitometric quantification of protein expression levels in (b). Expression was normalized against tubulin levels in (b). The Bax/Bcl_2_ ratio was calculated. Data are means ± SEM (*n* = 4 mice per group); *p < 0.05 and ***p < 0.001; RD vs HFrD in the same genotype, ^#^ *p* < 0.05 and ^##^ *p* < 0.01; *Cont.* vs *A/A* ^*Hep*^. (PDF 1081 kb)

Additional file 9: The levels of eIF2α phosphorylation in the livers of adult mice after 5 weeks of HFrD. Western blot analysis of liver lysates from 4-month-old *Cont.* and *A/A* ^*Hep*^ mice fed an RD or an HFrD for 5 wks. The * indicates non-specific bands in the Western blot. (PDF 325 kb)

Additional file 10: Transmission electron microscopy of hepatocytes in fructose-fed mice. Transmission electron microscopy (TEM) was performed on liver sections of 7-month-old *Cont.* and *A/A* ^*Hep*^ mice fed an RD or an HFrD for 16 wks. The arrowheads indicate the ER compartments. Representative images are shown (*n* = 3 mice per group). (PDF 1049 kb)

Additional file 11: A long-term high fructose diet causes altered expression of ROS-defense genes and induces ROS accumulation and oxidative damage in liver tissue of adult *A/A* ^*Hep*^ mice. (a) Quantitative real-time PCR analysis of the expression of selected mRNAs (ROS-defense genes) in liver tissues from 7-month-old *Cont.* and *A/A* ^*Hep*^ mice fed an RD or an HFrD for 16 wks. Data are shown as means ± SEM (*n* = 5 ~ 6 mice per group); *p < 0.05, **p < 0.01 and ***p < 0.001; RD vs HFrD for the same genotype, ^###^ *p* < 0.001; *Cont.* vs *A/A* ^*Hep*^ *,* ^&^ *p* < 0.05; *Cont.*(RD) vs *A/A* ^*Hep*^ (HFrD). (b) Western blot analysis of antioxidant proteins in liver tissues of 7-month-old *Cont.* and *A/A* ^*Hep*^ mice fed an RD or an HFrD for 16 wks. (c) Densitometric quantification of the protein levels in the panels in (b). Expression values are normalized against tubulin levels. (d) Hepatic GSH and GSSG levels in liver tissues from 7-month-old *Cont.* and *A/A* ^*Hep*^ mice fed an RD or an HFrD for 16 wks. Data are means ± SEM (*n* = 5 ~ 6 mice per group). (e) Fluorescence microscopic observations of frozen sections stained with dihydroethidium (DHE). Representative images are shown (*n* = 3 mice per group). Mean fluorescence intensity (MFI) of DHE staining was measured using image analysis software. Data are means ± SEM (*n* = 3 mice per group). (f) and (g) Immunohistochemistry of nitrotyrosine (f) and 4-HNE (g) in liver sections from 7-month-old *Cont.* and *A/A* ^*Hep*^ mice fed an HFrD for 16 wks. Inset shows a magnified view of the area outlined in the black dotted box. Measurements of nitrotyrosine and 4-HNE-positive cells are shown in the graphs. Data are means ± SEM (*n* = 6 mice per group); ^##^ *p* < 0.01 and ^###^ *p* < 0.001. (PDF 3516 kb)

## Abbreviations

ALT: Alanine transaminase
AST: Aspartate transaminase
DHE: Dihydroethidine hydrochloride
DNL: de novo lipogenesis
EIC: Extracted ion chromatogram
eIF2α: Eukaryotic translation initiation factor 2 alpha subunit
ER: Endoplasmic reticulum
ERAD: Endoplasmic-reticulum-associated protein degradation
GSH: Glutathione
GSSG: Oxidized GSH
H&E: Hematoxylin and eosin
HCC: Hepatocellular carcinoma
HFrD: High fructose diet
KHK: Ketohexokinase
MRM: Multiple reaction monitoring
NAFLD: Nonalcoholic fatty liver disease
NAS: nafld activity score
NASH: Nonalcoholic steatohepatitis
ROS: Reactive oxygen species
TEM: Transmission electron microscope
Tm: Tunicamycin
UPR: Unfolded protein respones
