# Supplementary figures and images for: eIF2α phosphorylation is required to prevent hepatocyte death and liver fibrosis in mice challenged with a high fructose diet
Source: Nutr Metab (Lond). 2017 Aug 1;14:48. doi: 10.1186/s12986-017-0202-6 (PMC5537942; doi:10.1186/s12986-017-0202-6)

**A**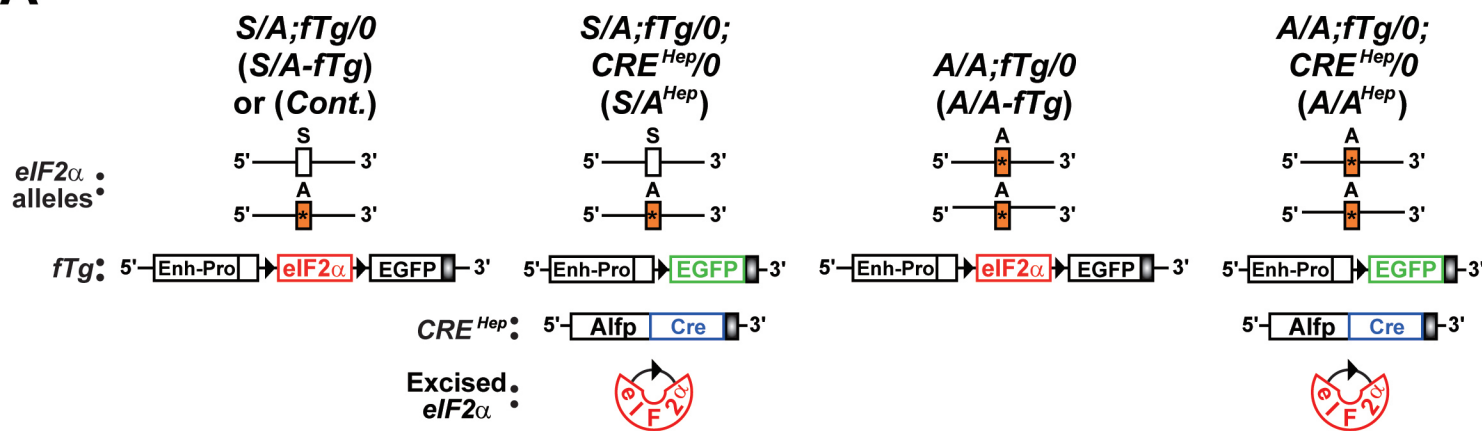**B**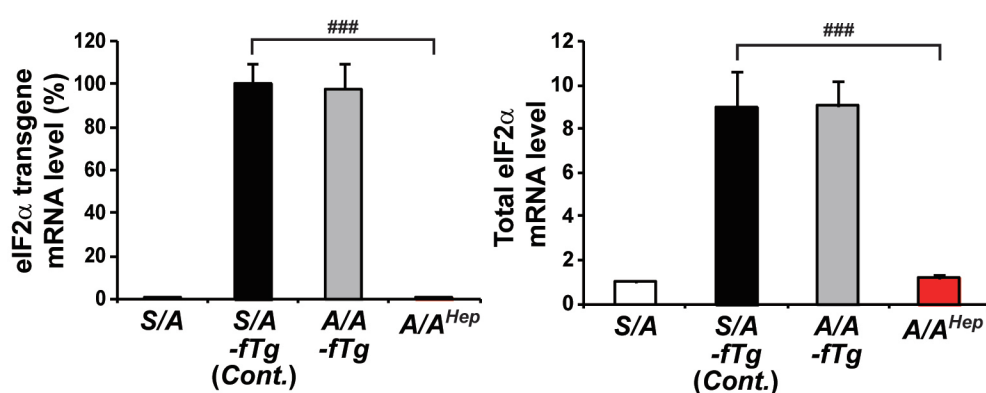**C**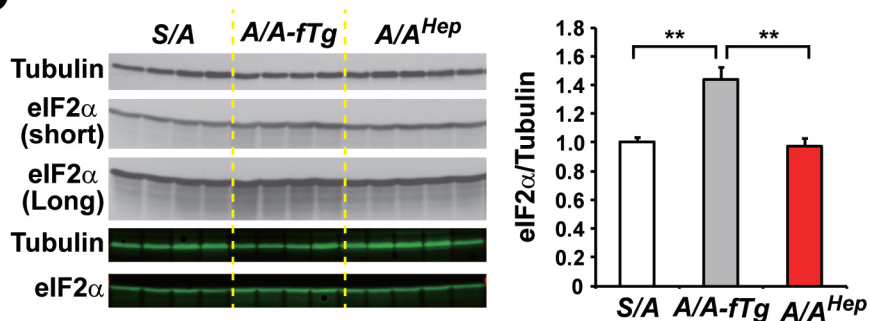**D**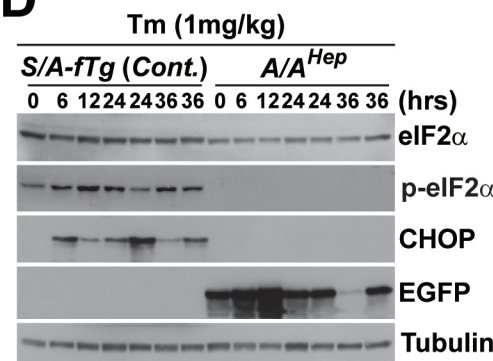**E**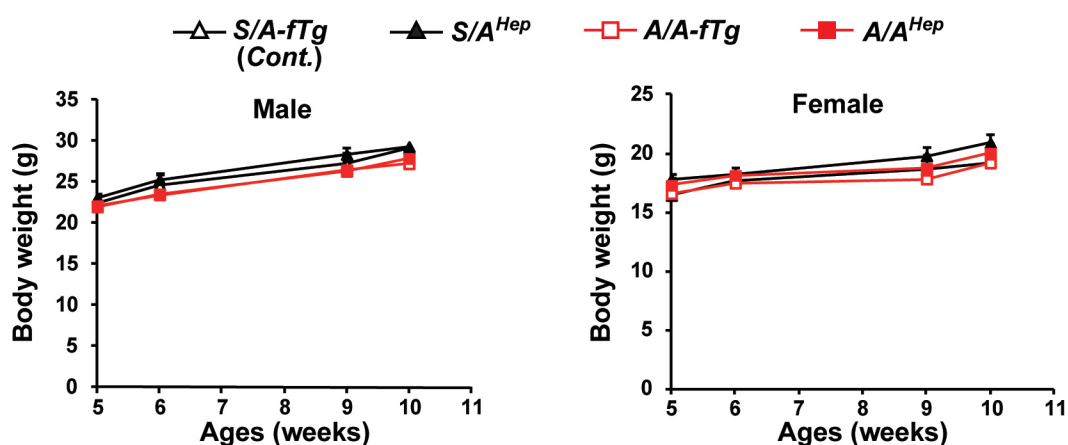

Supplement: Supplementary file 1 — eIF2α phosphorylation in hepatocytes is dispensable for survival of adult mice. (a) Diagram depicting the four genotypes of mice used in these experiments. S/A and A/A represent heterozygous and homozygous eIF2α Ser51Ala (*) mutation(s) in exon 2 of one eIF2α allele and both eIF2α alleles, respectively. fTg/0 represents the floxed wild type (WT) eIF2α transgene driven by the CMV enhancer and chicken β-actin promoter (Enh-Pro). The loxP sequences (black arrowheads) allow excision of the WT eIF2α floxed transgene (fTg) and expression of EGFP, an indicator of recombination. CRE Hep /0 represents the Cre recombinase transgene driven by the promoter (Alfp) of Alb1 (encoding albumin) and the enhancer of Afp (encoding alpha-fetoprotein). (b) Efficiency of deletion of the fTg in liver tissues. Results from quantitative RT-PCR analyses of transgenic and total eIF2α mRNAs are shown. Data are means ± SEM (n = 4 ~ 5 mice per group); ### p < 0.001; Cont. vs A/A Hep. (c) Western blot analysis of eIF2α protein expression driven by the fTg in liver tissues. To quantity expression of eIF2α, blots were incubated with anti-eIF2α antibody followed by IRDye-800 goat anti-rabbit IgG (LI-COR). Membranes were scanned on an Odyssey scanner (LI-COR) (lower two panels in left panels) and quantified with the Odyssey Software package. (d) Western blot analysis of liver lysates in Cont. and A/A Hep mice at the indicated times after Tm injection. Cont. mice and A/A Liv mice were injected with vehicle or tunicamycin (Tm, 1 mg/kg). (e) Body weight measurements of fTg -deleted A/A Liv mice. At the weeks, body weight was measured in both male and female mice. Data are means ± SEM (n = 6-14 mice per group). (PDF 1987 kb) [file 12986_2017_202_MOESM1_ESM.pdf]

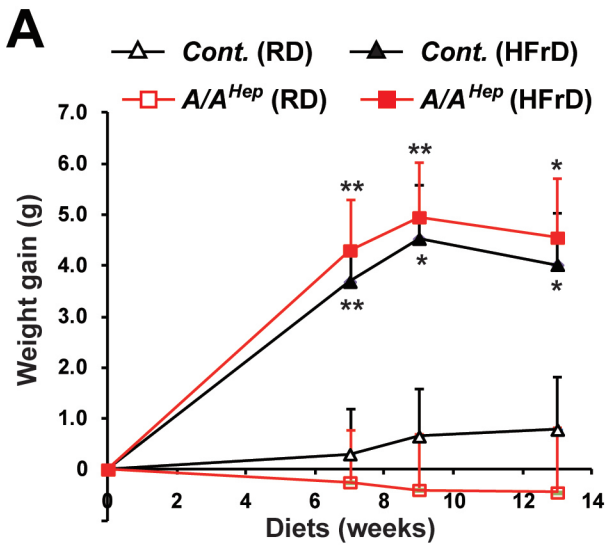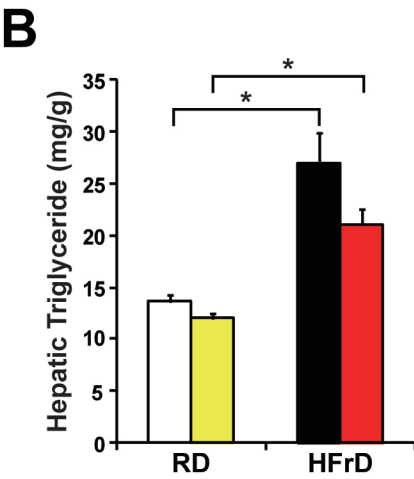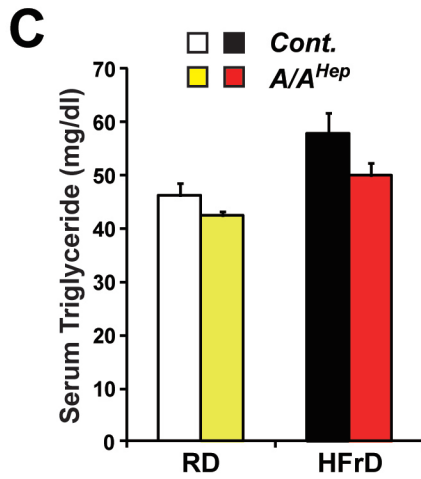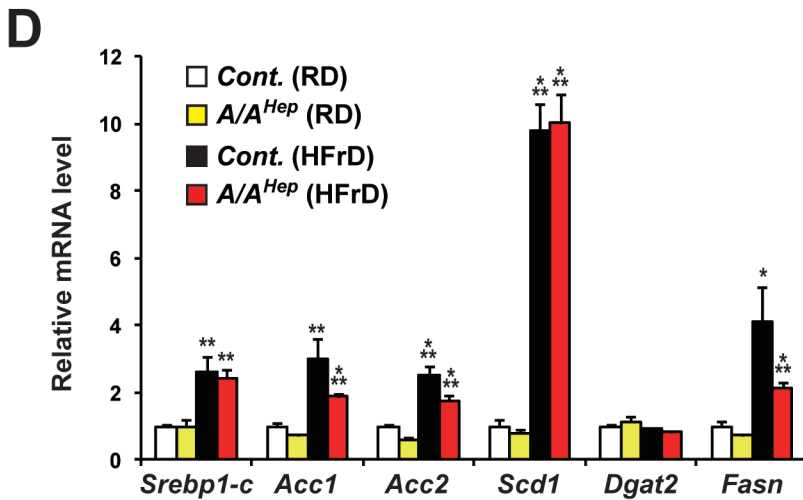

Supplement: Supplementary file 5 — Comparison of hepatic and serum triglyceride levels in RD and HFrD-fed mice. (a) Weight gain of Cont. and A/A Hep mice on an RD or an HFrD for 13 wks. Data are means ± SEM (n = 6 mice per group); *p < 0.05 and **p < 0.01; RD vs HFrD in the same genotype. (b) Hepatic and (c) serum triglyceride levels of Cont. and A/A Liv mice fed an RD or an HFrD for 16 wks. Data are means ± SEM (n = 6 mice per group); *p < 0.05; RD vs HFrD. (d) Quantitative real-time PCR analysis of expression of selected genes (fatty acid synthesis and metabolism) in liver lysates from Cont. and A/A Hep mice fed an RD or an HFrD for 16 wks. Data are means ± SEM (n = 6 mice per group); *p < 0.05, **p < 0.01 and ***p < 0.001; RD vs HFrD. (PDF 929 kb) [file 12986_2017_202_MOESM5_ESM.pdf]

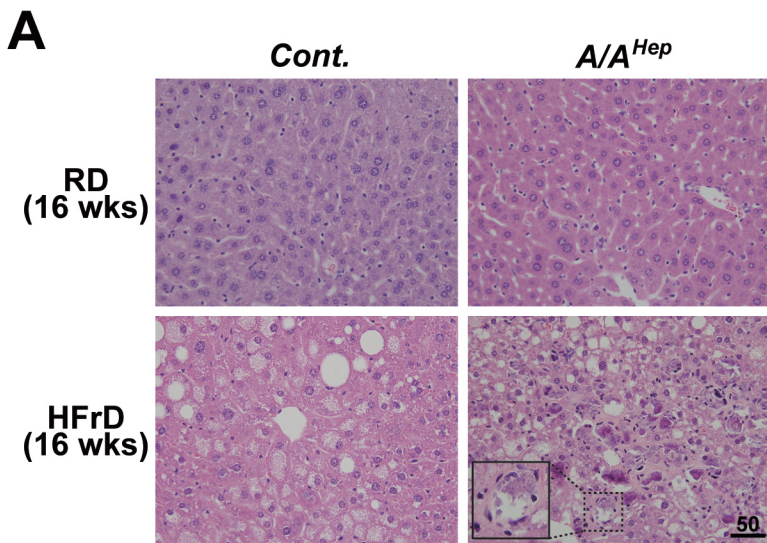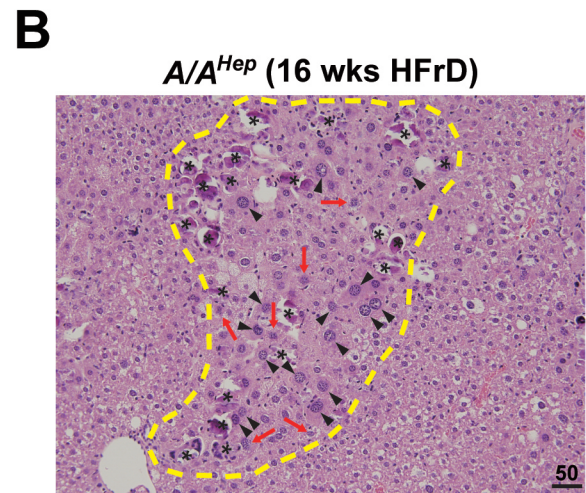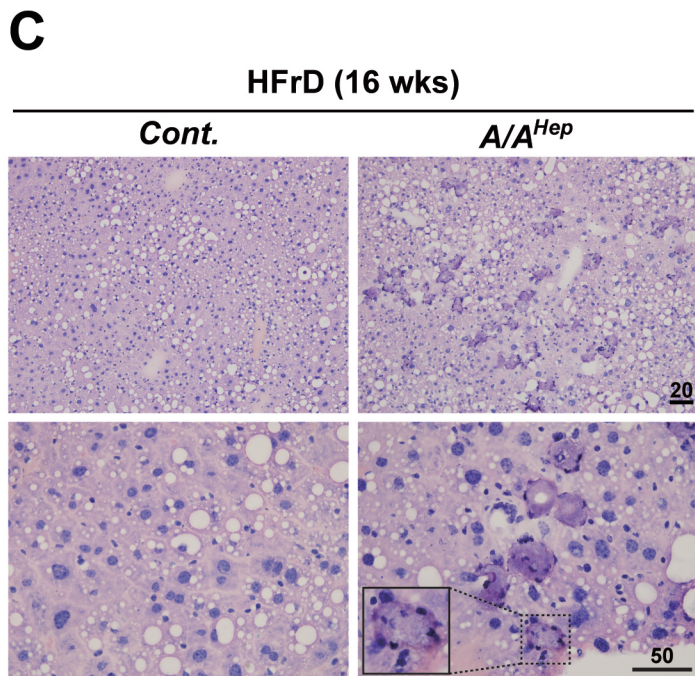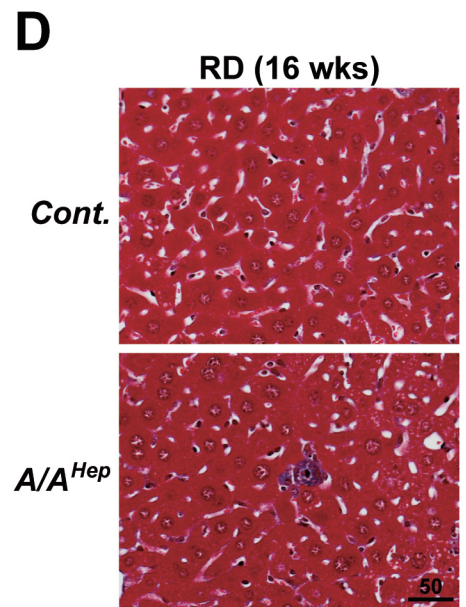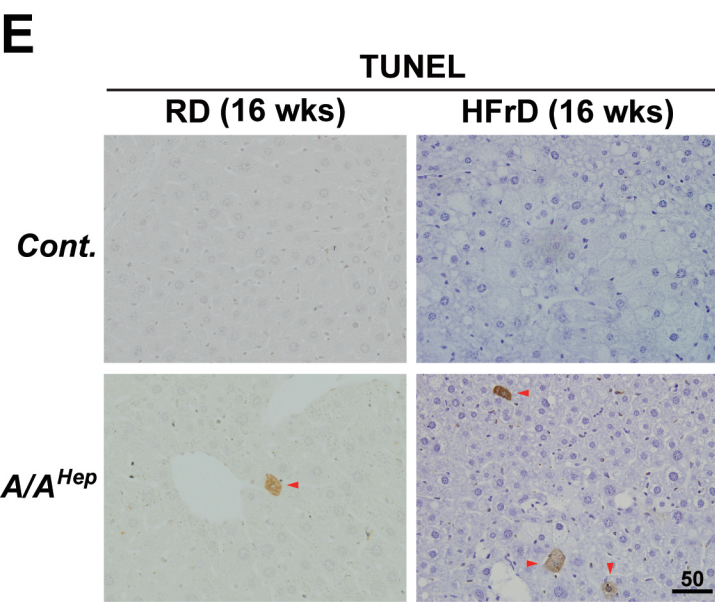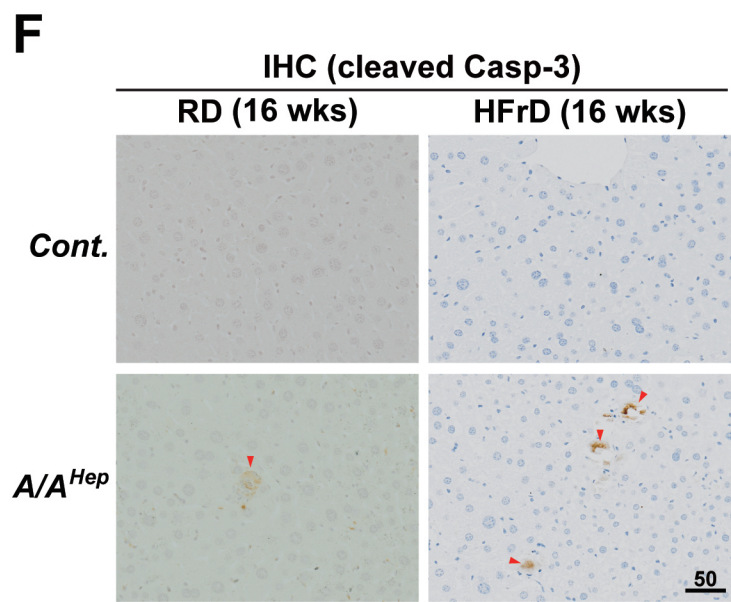

Supplement: Supplementary file 6 — eIF2α phosphorylation in adult mice prevents hepatocyte death induced by a long-term HFrD. (a) Hematoxylin and eosin (H&E)-stained images of paraffin-embedded liver sections from 7-month-old Cont. and A/A Hep mice fed an RD or an HFrD for 16 wks. Inset shows a magnified view of the area outlined in the black dotted box. The image reveals a necrotic hepatocyte surrounded by leukocytes. (b) Hematoxylin and eosin (H&E)-stained images of liver tissue sections from 7-month-old A/A Liv mice fed an HFrD for 16 wks. Dashed lines delineate the area consisting of ruptured dead cells (asterisks) and dying hepatocytes with fragmented nuclei (red arrow) and atypic nuclei (arrow head). (c) Hematoxylin and eosin (H&E)-stained images of OCT-embedded liver sections from 7-month-old Cont. and A/A Hep mice fed an RD or an HFrD for 16 wks. To prepare frozen blocks, tissue was embedded in Tissue-Tek OCT compound (Sakura Finetek). OCT-embedded sections (7 μm) of frozen livers were stained with haematoxylin and eosin (H&E). Inset shows a magnified view of the area outlined in the black dotted box. The image reveals a necrotic hepatocyte surrounded by leukocytes. (d) Masson’s trichrome-stained images of liver tissue sections from 7-month-old Cont. and A/A Hep mice fed an RD for 16 wks. Representative images are shown (n = 6 mice per group). (e) TUNEL analysis and (f) Immunohistochemical staining of cleaved caspase3 of liver tissue sections from 7-month-old Cont. and A/A Hep mice fed an RD or an HFrD for 16 wks. The arrowheads indicate positive cells. Representative images are shown (n = 6 mice per group). (PDF 6082 kb) [file 12986_2017_202_MOESM6_ESM.pdf]

**A**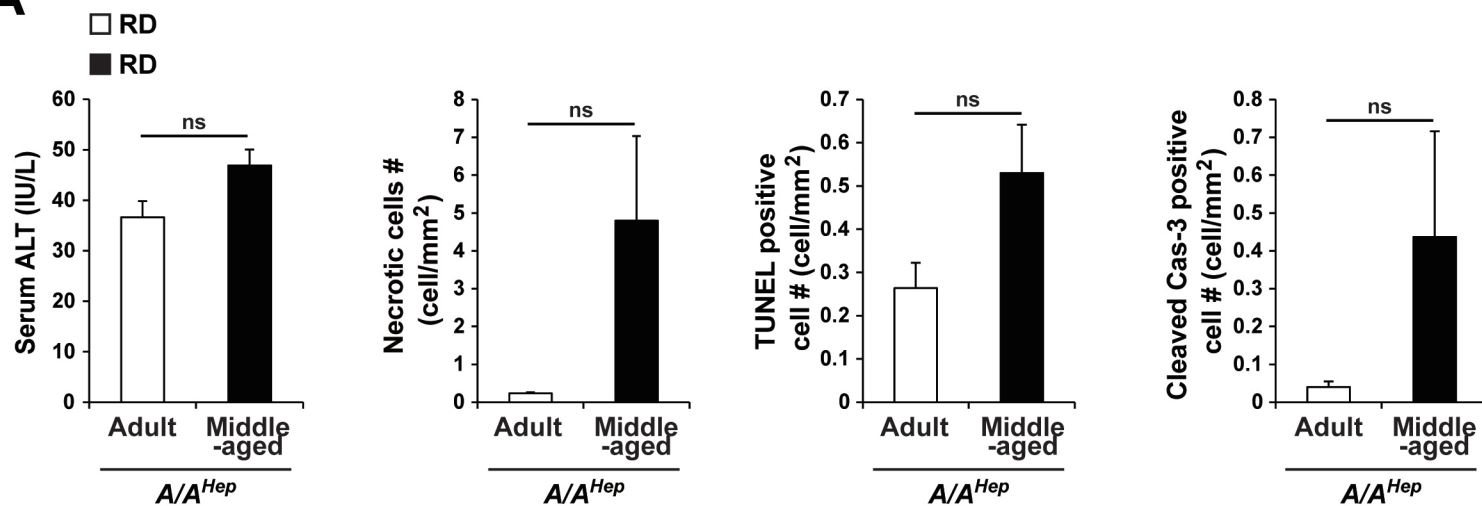**B**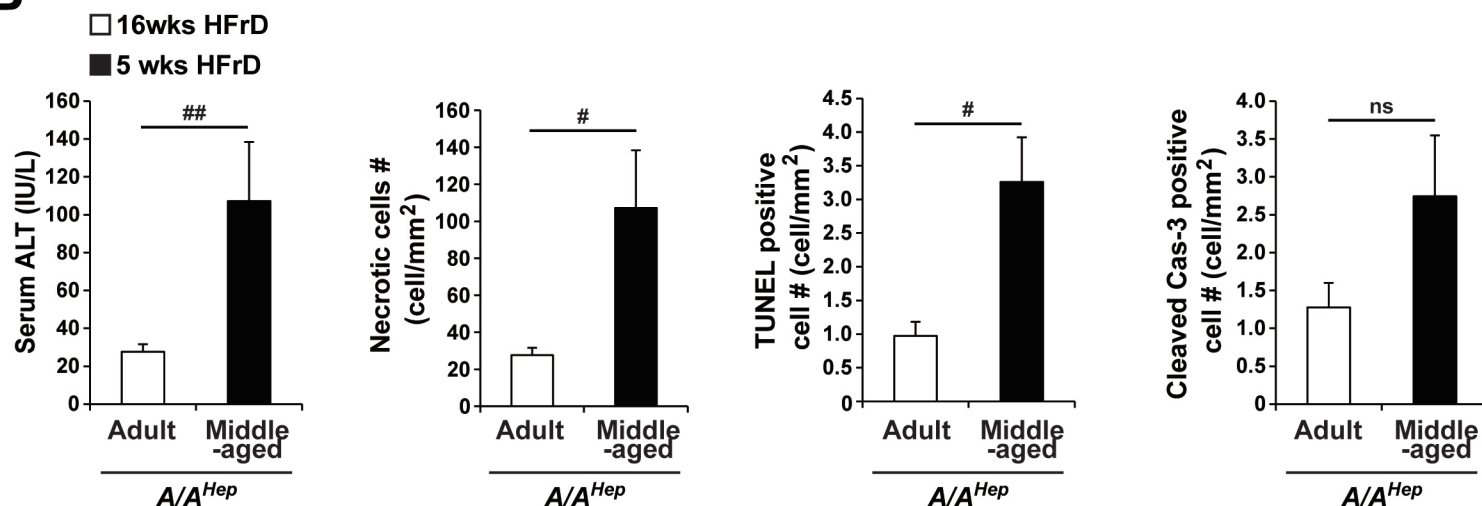

Supplement: Supplementary file 7 — The severity of HFrD-mediated damage is higher in middle-aged A/A Hep mice. (a) The data (serum ALT level, necrotic cell #, TUNEL positive cell #, and cleaved caspase 3 positive cell # of RD-fed adult A/A Hep mice from Fig. 3a–d and RD-fed middle-aged A/A Hep mice from Fig. 2b–e) were reused to generate the graphs. The data were statistically analyzed to evaluate the difference of measured values. Data are means ± SEM (n = 6 mice for RD-fed adult A/A Hep mice and n = 8 mice for RD-fed middle-aged A/A Hep mice). ns stands for no significant. (b) The data (serum ALT level, necrotic cell #, TUNEL positive cell #, and cleaved caspase 3 positive cell # of 16 wks HFrD-fed adult A/A Hep mice from Fig. 3a–d and 5 wks HFrD-fed middle-aged A/A Hep mice from Fig. 2b–e) were reused to generate the graphs. The data were statistically analyzed to evaluate the difference of measured values. Data are means ± SEM (n = 6 mice for 16 wks HFrD-fed adult A/A Hep mice and n = 7 mice for 5 wks HFrD-fed middle-aged A/A Hep mice); # p < 0.05 and ## p < 0.01; Adult vs Middle-aged. ns stands for no significant. (PDF 1137 kb) [file 12986_2017_202_MOESM7_ESM.pdf]

**A**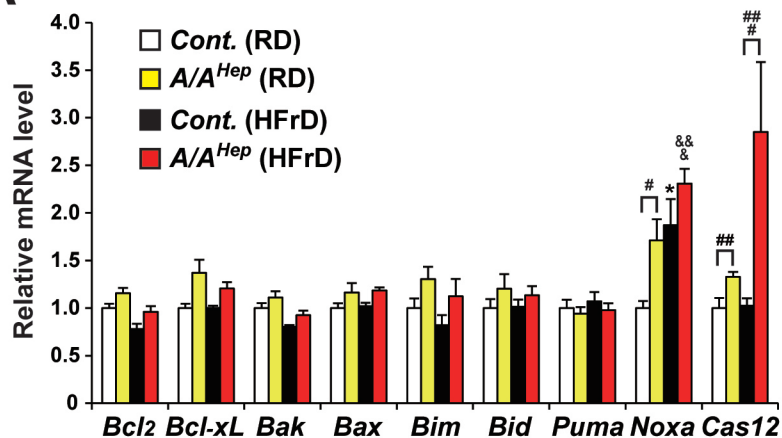**B**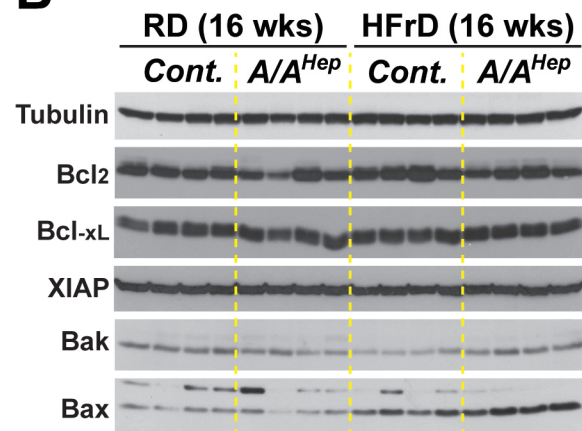**C**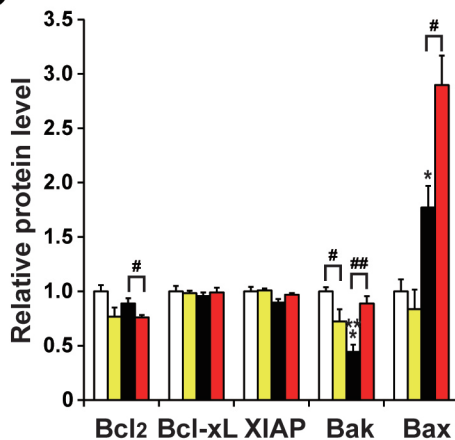**D**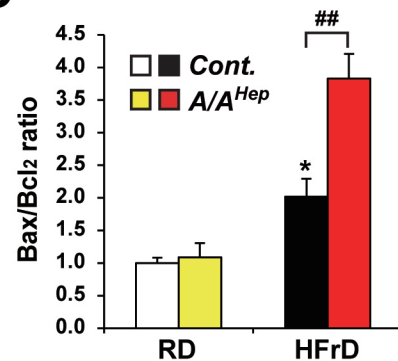

Supplement: Supplementary file 8 — A high fructose diet increases expression of proapoptotic genes in liver tissues of A/A Hep mice. (a) Quantitative real-time PCR analysis to assess the expression of selected genes (anti/proapoptotic genes) in liver tissues from 7-month-old Cont. and A/A Hep mice fed an RD or an HFrD for 16 wks. Data are means ± SEM (n = 5 ~ 6 mice per group); *p < 0.05 and **p < 0.01; RD vs HFrD in the same genotype, # p < 0.05, ## p < 0.01 and ### p < 0.001; Cont. vs A/A Hep , & p < 0.05 and &&& p < 0.001; Cont.(RD) vs A/A Hep(HFrD). (b) Western blot analysis of selected anti/proapoptotic proteins in liver lysates from 7-month-old Cont. and A/A Hep mice fed an RD or an HFrD for 16 wks. (c) and (d) Densitometric quantification of protein expression levels in (b). Expression was normalized against tubulin levels in (b). The Bax/Bcl2 ratio was calculated. Data are means ± SEM (n = 4 mice per group); *p < 0.05 and ***p < 0.001; RD vs HFrD in the same genotype, # p < 0.05 and ## p < 0.01; Cont. vs A/A Hep. (PDF 1081 kb) [file 12986_2017_202_MOESM8_ESM.pdf]

# Choi et al. Additional file 9

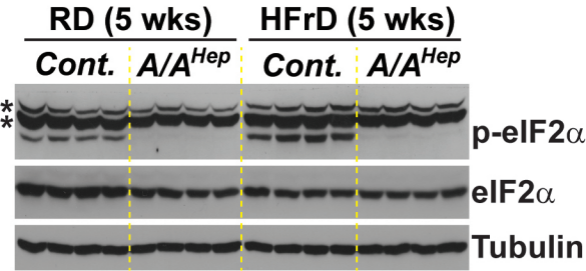

Supplement: Supplementary file 9 — The levels of eIF2α phosphorylation in the livers of adult mice after 5 weeks of HFrD. Western blot analysis of liver lysates from 4-month-old Cont. and A/A Hep mice fed an RD or an HFrD for 5 wks. The * indicates non-specific bands in the Western blot. (PDF 325 kb) [file 12986_2017_202_MOESM9_ESM.pdf]

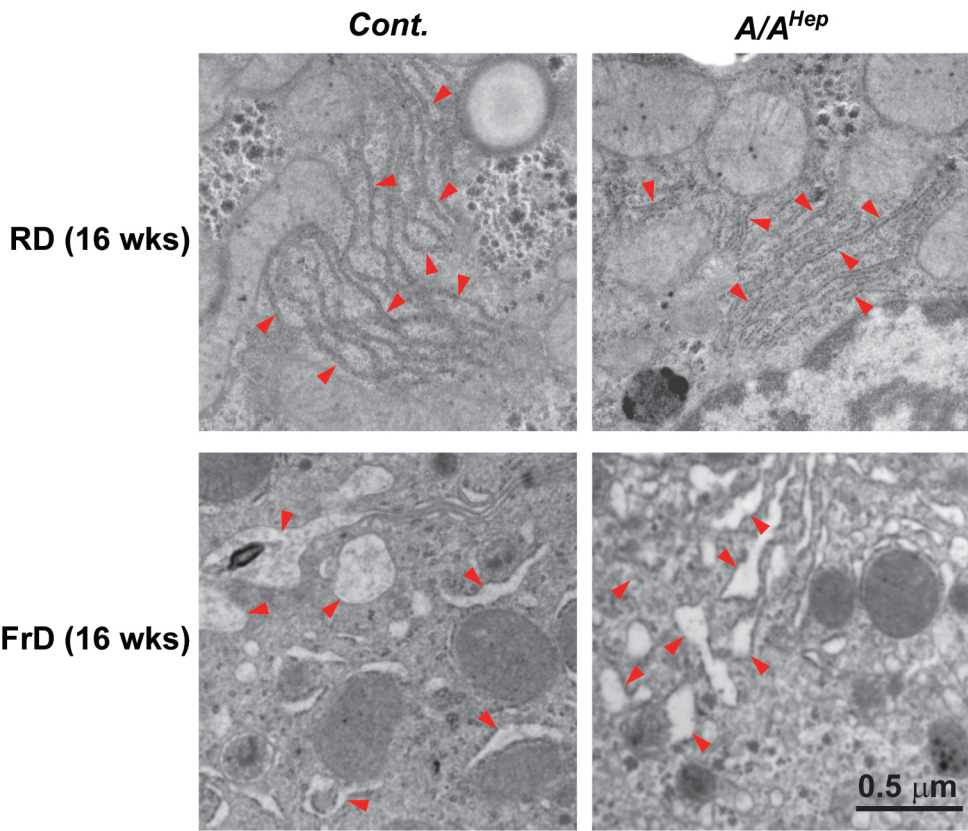

Supplement: Supplementary file 10 — Transmission electron microscopy of hepatocytes in fructose-fed mice. Transmission electron microscopy (TEM) was performed on liver sections of 7-month-old Cont. and A/A Hep mice fed an RD or an HFrD for 16 wks. The arrowheads indicate the ER compartments. Representative images are shown (n = 3 mice per group). (PDF 1049 kb) [file 12986_2017_202_MOESM10_ESM.pdf]

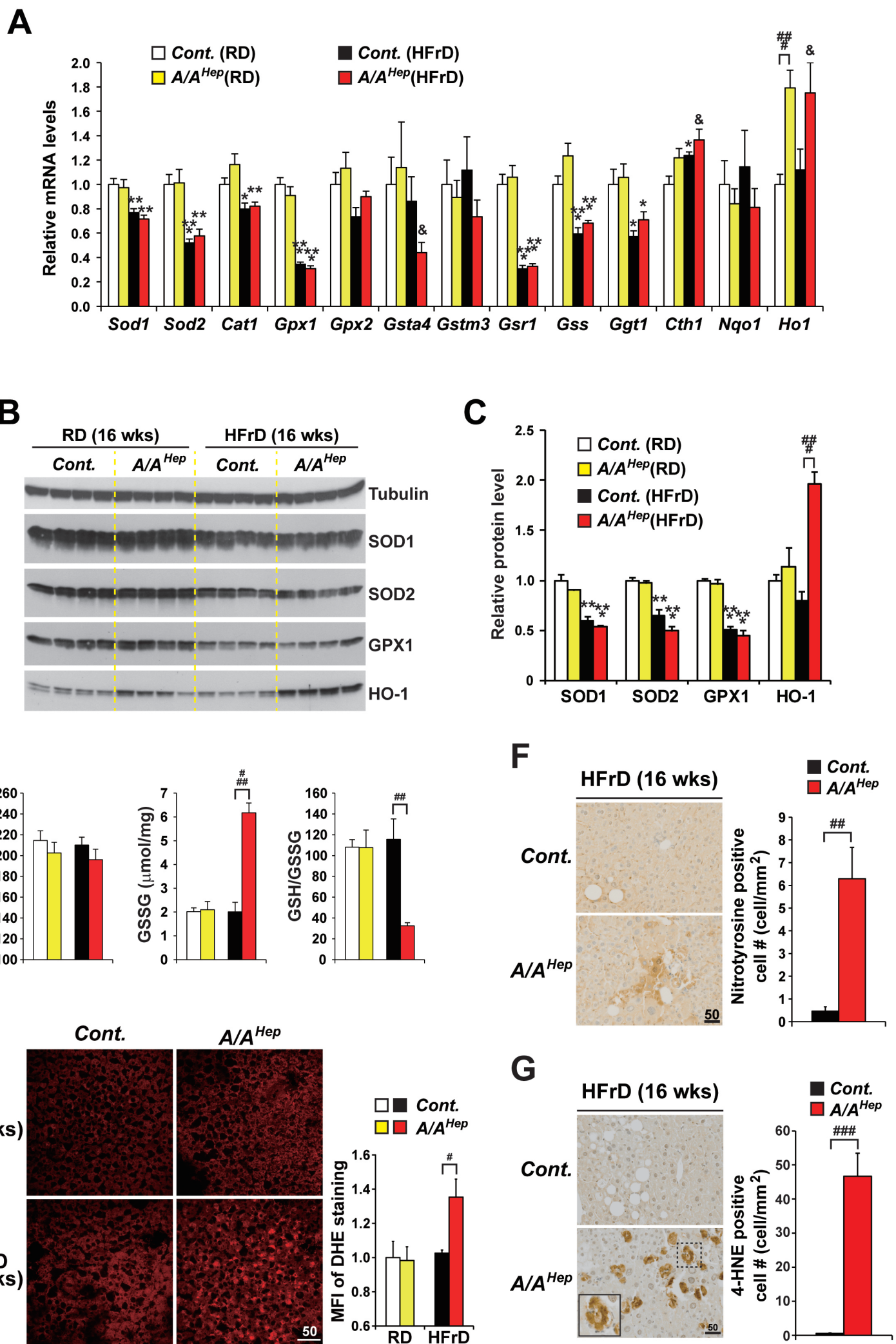

Supplement: Supplementary file 11 — A long-term high fructose diet causes altered expression of ROS-defense genes and induces ROS accumulation and oxidative damage in liver tissue of adult A/A Hep mice. (a) Quantitative real-time PCR analysis of the expression of selected mRNAs (ROS-defense genes) in liver tissues from 7-month-old Cont. and A/A Hep mice fed an RD or an HFrD for 16 wks. Data are shown as means ± SEM (n = 5 ~ 6 mice per group); *p < 0.05, **p < 0.01 and ***p < 0.001; RD vs HFrD for the same genotype, ### p < 0.001; Cont. vs A/A Hep , & p < 0.05; Cont.(RD) vs A/A Hep (HFrD). (b) Western blot analysis of antioxidant proteins in liver tissues of 7-month-old Cont. and A/A Hep mice fed an RD or an HFrD for 16 wks. (c) Densitometric quantification of the protein levels in the panels in (b). Expression values are normalized against tubulin levels. (d) Hepatic GSH and GSSG levels in liver tissues from 7-month-old Cont. and A/A Hep mice fed an RD or an HFrD for 16 wks. Data are means ± SEM (n = 5 ~ 6 mice per group). (e) Fluorescence microscopic observations of frozen sections stained with dihydroethidium (DHE). Representative images are shown (n = 3 mice per group). Mean fluorescence intensity (MFI) of DHE staining was measured using image analysis software. Data are means ± SEM (n = 3 mice per group). (f) and (g) Immunohistochemistry of nitrotyrosine (f) and 4-HNE (g) in liver sections from 7-month-old Cont. and A/A Hep mice fed an HFrD for 16 wks. Inset shows a magnified view of the area outlined in the black dotted box. Measurements of nitrotyrosine and 4-HNE-positive cells are shown in the graphs. Data are means ± SEM (n = 6 mice per group); ## p < 0.01 and ### p < 0.001. (PDF 3516 kb) [file 12986_2017_202_MOESM11_ESM.pdf]
