# Supplementary material for: eIF2α phosphorylation is required to prevent hepatocyte death and liver fibrosis in mice challenged with a high fructose diet
Source: Nutr Metab (Lond). 2017 Aug 1;14:48. doi: 10.1186/s12986-017-0202-6 (PMC5537942; doi:10.1186/s12986-017-0202-6)
Supplement: Supplementary file 4 — Hepatocyte death and fibrosis in 13 month-old A/A Hep mice fed a regular diet (RD) or a 60% high fructose diet (HFrD) for 5 wks (a) Hematoxylin and eosin (H&E)-stained images of liver tissue sections from 13-month-old Cont. (A/A-fTg) (n = 5) and A/A Hep mice (n = 8) fed an RD. Representative images are shown. (b) TUNEL and (c) Cleaved caspase-3-stained images of liver tissue sections from 13-month-old Cont. (A/A-fTg or S/A-fTg) and A/A Hep mice fed an RD (n = 5 ~ 8 mice per group) or an HFrD (n = 7 mice per group) for 5 wks. The arrowheads indicate positive cells. Representative images are shown. (d) Sirius red-stained and (e) Masson’s trichrome-stained images of liver tissue sections from 13-month-old Cont. (A/A-fTg) (n = 5) and A/A Hep mice (n = 8) fed an RD. Representative images are shown. (PDF 5270 kb) [file 12986_2017_202_MOESM4_ESM.pdf]

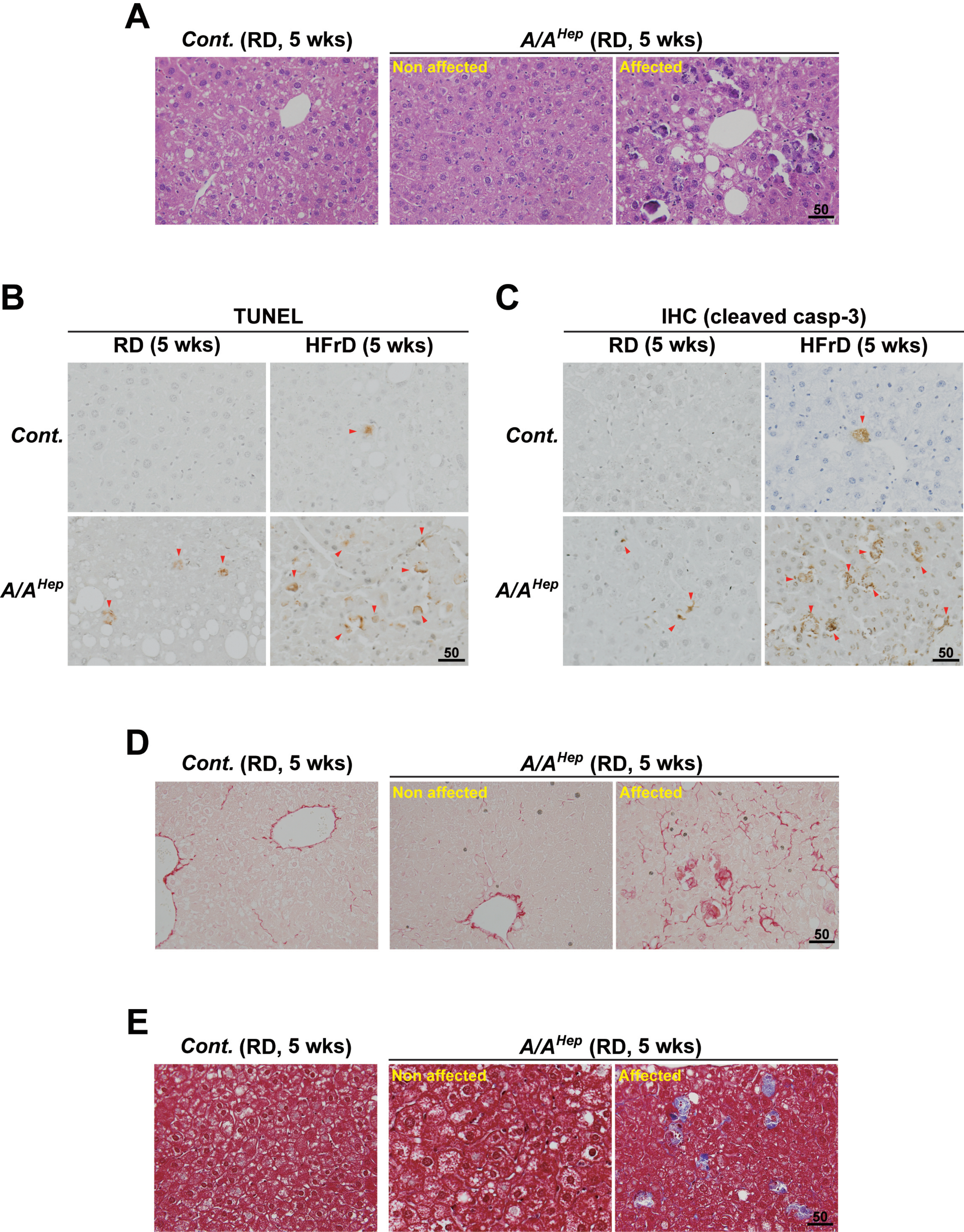
A
Cont. (RD, 5 wks)
A/A^Hep (RD, 5 wks)
Non affected
Affected
50
B
TUNEL
RD (5 wks)
HFrD (5 wks)
Cont.
A/A^Hep
50
C
IHC (cleaved casp-3)
RD (5 wks)
HFrD (5 wks)
Cont.
A/A^Hep
50
D
Cont. (RD, 5 wks)
A/A^Hep (RD, 5 wks)
Non affected
Affected
50
E
Cont. (RD, 5 wks)
A/A^Hep (RD, 5 wks)
Non affected
Affected
50
